# Determinants of post COVID-19 clinic attendance among SARS-CoV-2-infected individuals in Stockholm, Sweden: a population-based cohort study

**DOI:** 10.1136/bmjopen-2024-098344

**Published:** 2025-06-17

**Authors:** Pontus Hedberg, Peder af Geijerstam, John Karlsson Valik, Christer Almgren-Lidman, Anders Ternhag, Pontus Naucler

**Affiliations:** 1 Department of Medicine Huddinge, Karolinska Institutet, Stockholm, Sweden; 2 Primary Health Care Center Cityhälsan Centrum, and Department of Health, Medicine and Caring Sciences, Linköping University, Linköping, Sweden; 3 Department of Medicine Solna, Karolinska Institutet, Stockholm, Sweden; 4 Department of Infectious Diseases, Karolinska University Hospital, Stockholm, Sweden

**Keywords:** COVID-19, SARS-CoV-2 Infection, Post-Acute COVID-19 Syndrome

## Abstract

**Abstract:**

**Objectives:**

Investigate determinants of post-COVID-19 condition (PCC) clinic attendance among participants not hospitalised versus hospitalised during the SARS-CoV-2 infection.

**Design:**

Retrospective cohort study.

**Setting:**

Six population-based registers with high coverage to cover all adults residing in Stockholm County, Sweden.

**Participants:**

Adults residing in Stockholm County on 31 January 2020, with a SARS-CoV-2 infection through 30 November 2022, who did not die or move out of Stockholm County within 90 days.

**Primary outcome measures:**

PCC clinic attendance from 90 days after the SARS-CoV-2 test until date of death, date of moving out, or 30 November 30,2023.

**Results:**

Of non-hospitalised and hospitalised participants, 737 of 464 674 (0.2%) and 433 of 23 374 (1.9%), respectively, attended a PCC clinic. A total of 75 878 (16.3%) of non-hospitalised participants and 6190 (26.5%) of hospitalised participants presented with new-onset symptoms that could indicate PCC in primary care. The strongest determinants of attendance among non-hospitalised participants were mental health disorder (adjusted risk ratio (aRR) 2.57, 95% CI 2.21 to 2.98), asthma (2.39, 1.97–2.92) and >4 PCC symptoms in 2019 (2.27, 1.60–3.24), and among hospitalised participants were >31 sick days in 2019 (1.94, 1.47–2.56), 1–30 sick days in 2019 (1.56, 1.06–2.29) and obesity (1.51, 1.19–1.93). The most common clinical presentation was fatigue (n=526, 71.4%) among non-hospitalised and dyspnoea (n=148, 34.2%) among hospitalised participants.

**Conclusions:**

PCC clinic attendance characteristics differed between non-hospitalised and hospitalised participants. Distinguishing PCC from conditions with overlapping symptoms and determining the appropriate level of care may be challenging, with risk of resource displacement effects and inappropriate care.

**STRENGTHS AND LIMITATIONS OF THIS STUDY**

- Cross-linking of several population-based registers, which enabled us to not only identify visits at post-COVID-19 condition (PCC) clinics but also visits in primary care where a PCC diagnosis was registered.
- Access to not only all positive SARS-CoV-2 PCR tests, but also positive SARS-CoV-2 serology tests during 2020, we could include around 50 000 participants who had an infection during 2020, primarily non-hospitalised participants.
- Able to distinguish between participants who were not hospitalised versus hospitalised during the acute SARS-CoV-2 infection, which enabled us to demonstrate large differences in both factors influencing PCC clinic attendance and symptoms reported during those visits.
- The generalisability of these findings is difficult to assess due to a lack of comparable studies.
- This study was set in the capital region of a high-income country with publicly funded healthcare services, thus reducing selection bias. Our results might, however, not be generalisable to settings with different healthcare funding systems.

## Introduction

 A subset of individuals with a history of SARS-CoV-2 infection experiences persistent symptoms, a condition referred to as “long COVID” or post-COVID-19 condition (PCC). Specialised PCC clinics have been established, but services have been scaled back, and the pace of clinical trials to identify PCC treatments is insufficient, raising concerns among patients, advocacy groups and healthcare providers.^1 2^ Studies on individuals attending vs not attending PCC clinics are lacking, which hinders our understanding of PCC healthcare needs and potential disparities in service utilisation. Furthermore, many studies include only PCC clinic attendees, which may limit external validity if these individuals differ from others with prior SARS-CoV-2 exposure.

In Stockholm County, Sweden (population ~2.4 million), two PCC clinics (one at Karolinska University Hospital Huddinge (KUHH) opened in June 2021 and one at Danderyd Hospital (DH) opened in 2020) provide PCC-related healthcare services to the adult population.^3^ Both clinics receive patients referred by physicians, while the KUHH clinic also accepts self-referrals.^4^ Referral is indicated to the KUHH clinic for individuals with a new disability of at least 50% lasting >3 months and to the DH clinic for individuals with newly developed cognitive impairments and pronounced fatigue that affect activities including return to work for >3 months. In this study, we investigated determinants of attendance at either of these two PCC clinics among individuals who were not hospitalised and hospitalised during the acute SARS-CoV-2 infection, respectively.

## Methods

### Study design and data sources

This retrospective cohort study, approved by the Swedish Ethical Review Board, linked data from six population-based data sources: Stockholm regional healthcare data warehouse, SmiNet, Statistics Sweden, the Swedish Intensive Care Registry, the National Vaccination Register and the Quality Register for SARS-CoV-2 (COVID-19), as previously described.^5 6^

### Study population

Individuals who lived in Stockholm County on 31 January 2020, the date of the first SARS-CoV-2 case in Sweden, were considered for inclusion. To achieve adequate baseline data, including sociodemographic data and comorbidities, individuals who had not lived in Stockholm County since at least 1 January 2019 were excluded. Among the remaining individuals, those aged >18 years at the time of SARS-CoV-2 infection, defined as a positive PCR through 30 November 2022, or a positive serology through 26 December 2020 (COVID-19 vaccinations started on 27 December 2020), were included. PCC clinic attendance was assessed from 90 days after the first positive PCR or serology, and individuals who either died or moved out of Stockholm County before 90 days were excluded.

Hospitalisation was defined as a hospital admission with a first positive SARS-CoV-2 test at any time from 14 days before admission through the date of discharge, and a main or secondary ICD-10 diagnosis code U07.1 or U07.2 at discharge. For participants who only had a positive serology test, hospitalisation status was based exclusively on the hospital ICD-10 discharge diagnosis codes. Participants were categorised as those that were not hospitalised and hospitalised (with or without admission to the intensive care unit (ICU)) during the SARS-CoV-2 infection, to enable comparison of characteristics depending on the severity of the infection. We hypothesised that differences in characteristics were plausible because of different care pathways (referred vs self-referred), which could require different levels of health literacy.

### Participants with post-COVID-19 condition (PCC) symptoms in primary care

To understand determinants of PCC clinic attendance after a primary care presentation which could indicate PCC, such a cohort was defined. Individuals with any of 19 out of 25 symptoms included in the WHO PCC case definition (online supplemental table S1) registered in primary care from 28 through 180 days after the first positive PCR or serology within primary care, who did not have a history of the symptom(s) in 2019, were included.^7^ Symptoms were identified by registered ICD-10 codes. The six symptoms blurred vision, cognitive dysfunction, memory issues, menstrual problems, allergies and post-exertional malaise were excluded due to insufficient coverage in the available data as previously described.^5^

### Determinants

Determinants included sex, age, region of birth, education level, age-standardised income quartile, number of sick days in 2019, number of primary care visits, number of outpatient specialist care visits, inpatient care in 2019, comorbidities, number of PCC symptoms in the WHO PCC clinical case definition in 2019, COVID-19 vaccination status before the SARS-CoV-2 infection and SARS-CoV-2 variant period. The year 2019 was selected to achieve a complete 1 year pre-pandemic assessment period. COVID-19 vaccination status was categorised as unvaccinated, or as having received 1, 2, 3, 4 or 5 or more doses administered at least 14 days before the acute SARS-CoV-2 infection. We did not adjust for the exact timing between vaccination and infection (see online supplemental table S1 for more details).

### Outcome

The outcome was at least one visit to any of the two PCC clinics any time from 90 days after the first positive SARS-CoV-2 PCR or serology until date of death, date of moving out of Stockholm County or 30 November 2023, whichever occurred first. Thus, all participants could possibly be followed up until at least 1 year after the infection. The number of PCC clinic visits and the associated diagnosis codes were also analysed and categorised into disease/symptom specific groups (online supplemental table S2).

A PCC clinic was also established at Karolinska University Hospital Solna (KUHS) in April 2020. This clinic did not have a formal mandate to deliver PCC care from the governing body of healthcare in Stockholm County and ceased operations in November 2021.^8^ Visits to this clinic were included in a sensitivity analysis. Furthermore, a PCC diagnosis in primary care only was considered an additional outcome to better understand if participants with PCC diagnosed in primary care without a subsequent PCC clinic visit differed from participants attending a PCC clinic. All study variables, including both determinants and outcomes, are described in more detail in online supplemental table S1.

### Statistical methods

Continuous variables were presented as median (interquartile interval (IQI)), and categorical variables were reported as frequencies (percentages). Modified Poisson regression models were used to obtain unadjusted and adjusted risk ratios (RRs) with 95% CIs for PCC clinic attendance for each of the baseline characteristics. Possible confounders were selected individually for each investigated characteristic, based on subject-matter knowledge and pre-existing scientific evidence (online supplemental table S3). These regression models were also used for the cohort with any of 19 out of 25 symptoms included in the WHO PCC case definition, as well as the sensitivity analysis also including visits to the PCC clinic at KUHS. To investigate the effect of death as a competing event among hospitalised participants, results from the adjusted modified Poisson regression models were compared with results from adjusted Fine-Gray subdistribution hazard models in a post-hoc analysis.^9^

Furthermore, comparisons were made between participants that attended a PCC clinic and those that received a PCC diagnosis in primary care only. χ2 tests were used for categorical variables and Mann-Whitney U tests were used for continuous variables.

Low levels of missing data were observed for region of birth, education level, age-standardised income quartile and number of sick days in 2019 (online supplemental table S1). Participants with missing data were excluded from analyses considering these variables. Data for all other variables were complete. An alpha level of 0.05 was used for all analyses. Analyses were conducted using R V.4.1.0 (R Core Team, Vienna, Austria) and R Studio V.2024.04.1 (Posit Software, Boston, MA, USA).

### Role of the funding source

The work was supported by the Swedish Research Council (Dnr 2021–04809 and Dnr 2021–06540) and the EuCARE Project funded by the European Union’s Horizon Europe Research and Innovation Programme under Grant Agreement No 101 046 016. The funder of the study had no role in study design, data collection, data analysis, data interpretation or writing of the report.

### Patient and public involvement

Patients or members of the general public were not involved in planning, recruitment or conduct of the study as such.

## Results

**Figure 1:**
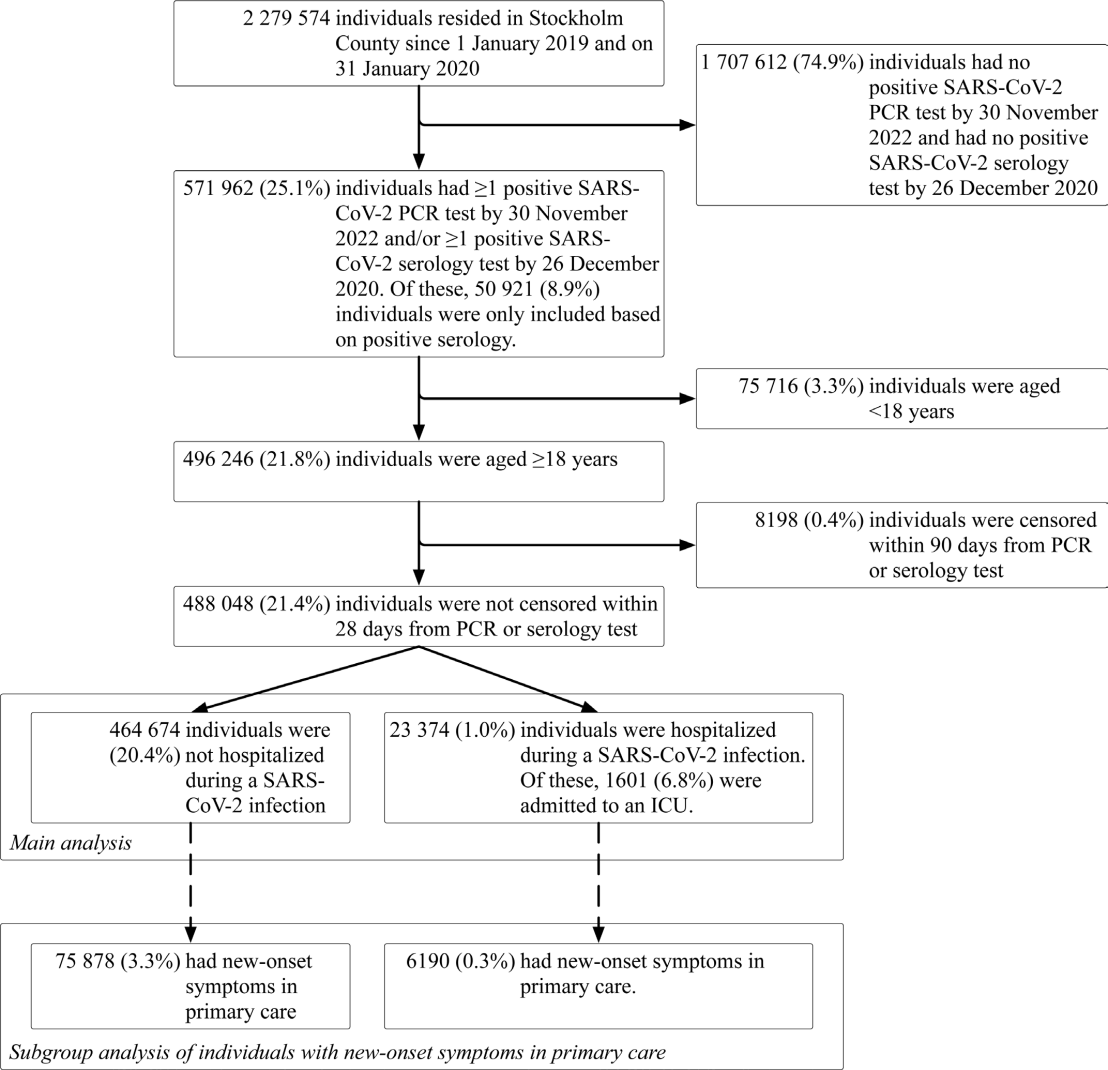
Study flow chart. July typically shows reduced healthcare utilisation in Sweden due to annual summer holidays for both patients and clinicians. ICU, Intensive care unit; PCR, PCR chain reaction; SARS-CoV-2, severe acute respiratory syndrome coronavirus 2.

A total of 2 279 574 individuals residing in Stockholm County on 31 January 2020 were considered for inclusion (figure 1). The final study population consisted of 488 048 adult participants who had tested positive for SARS-CoV-2 by PCR through 30 November 2022 or by serology through 26 December 2020 (48 887 (10.0%) participants included based on this criterion). Of these, 23 374 (4.8%) were hospitalised during the acute infection, of which 1601 (6.8%) were admitted to an ICU. A total of 75 878 (16.3%) of the non-hospitalised participants and 6190 (26.5%) of the hospitalised participants were included in the cohort with new-onset symptoms that could indicate PCC in primary care.

### Post-COVID-19 condition (PCC) clinic attendance

The number (percentage) attending a PCC clinic was 1170 (0.2%), being 737 (0.2%) among non-hospitalised and 433 (1.9%) among hospitalised participants. By SARS-CoV-2 infection variant period, the number of participants that attended a PCC clinic was 701 (0.4%) for Wild type, 265 (0.3%) for Alpha, 67 (0.2%) for Delta and 137 (0.1%) for Omicron. Among hospitalised participants, a decrease in the number of PCC clinic visits was observed from October 2022, whereas November 2022 was the calendar month with most visits from non-hospitalised participants (online supplemental figure S1). Of 292 participants that attended the PCC clinic at KUHS, but did not attend the PCC clinic at KUHH or DH, 278 (95%) were infected during the Wild type period, and 257 (88%) were hospitalised during the acute SARS-CoV-2 infection.

### Baseline characteristics

Of those that attended a PCC clinic, 548 (74.4%) of non-hospitalised compared with 173 (40.0%) of hospitalised participants were female (table 1). Among non-hospitalised participants, education level was higher in participants who attended a PCC clinic compared with those who did not attend, a difference not observed among hospitalised participants. Pre-existing mental health disorders were more common among non-hospitalised participants that attended (376 participants, 51.0%) versus did not attend (120 140 participants, 25.9%). No such differences were observed among hospitalised participants (28.9% vs 26.5%). Among those that attended a PCC clinic, non-hospitalised participants had numerically more visits (median 7, IQI 4–13) compared with hospitalised participants (median 3, IQI 1–7). Five participants (0.4%) attending a PCC clinic died during follow-up, compared with 7752 participants (1.6%) not attending a PCC clinic.

**Table 1:** Characteristics of participants by COVID-19 severity and PCC clinic attendance

	Not hospitalised (n=464 674)		Hospitalised (n=23 374)	
	No visit (n=463 937)	>1 visit (n=737)	No visit (n=22 941)	>1 visit (n=433)
Female sex	253 676 (54.7)	548 (74.4)	10 365 (45.2)	173 (40.0)
Age, years	42.0 (32.0, 53.0)	46.0 (39.0, 52.0)	67.0 (53.0, 79.0)	56.0 (48.0, 64.0)
Age category, years				
18–29	90 635 (19.5)	53 (7.2)	789 (3.4)	9 (2.1)
30–39	113 045 (24.4)	142 (19.3)	1716 (7.5)	28 (6.5)
40–49	113 506 (24.5)	293 (39.8)	2303 (10.0)	88 (20.3)
50–59	86 041 (18.5)	193 (26.2)	3585 (15.6)	139 (32.1)
60–69	37 485 (8.1)	51 (6.9)	3981 (17.4)	115 (26.6)
70–79	14 267 (3.1)	5 (0.7)	4862 (21.2)	50 (11.5)
>80	8958 (1.9)	0	5705 (24.9)	4 (0.9)
Born in Sweden*	336 454 (72.7)	588 (79.8)	13 764 (60.0)	224 (51.7)
Education level†				
Primary	57 166 (12.6)	33 (4.5)	5396 (24.3)	94 (22.2)
Secondary	164 922 (36.3)	235 (32.0)	9127 (41.2)	181 (42.8)
Tertiary	232 170 (51.1)	467 (63.5)	7640 (34.5)	148 (35.0)
Age-standardised income quartile‡				
First	79 449 (17.2)	150 (20.4)	7470 (32.6)	133 (30.7)
Second	122 924 (26.6)	223 (30.3)	6167 (26.9)	139 (32.1)
Third	129 556 (28.0)	228 (30.9)	4968 (21.7)	100 (23.1)
Fourth	130 686 (28.2)	136 (18.5)	4320 (18.8)	61 (14.1)
Number of sick days in 2019‡				
0	420 973 (91.0)	556 (75.4)	21 098 (92.0)	343 (79.2)
1–30	18 749 (4.1)	64 (8.7)	603 (2.6)	27 (6.2)
>31	22 893 (4.9)	117 (15.9)	1224 (5.3)	63 (14.5)
Primary care visits in 2019				
0	188 221 (40.6)	182 (24.7)	4746 (20.7)	95 (21.9)
1–2	170 819 (36.8)	272 (36.9)	7676 (33.5)	144 (33.3)
3–4	61 608 (13.3)	147 (19.9)	4573 (19.9)	82 (18.9)
>5	43 289 (9.3)	136 (18.5)	5946 (25.9)	112 (25.9)
Outpatient specialist care in 2019				
0	241 586 (52.1)	232 (31.5)	6847 (29.8)	148 (34.2)
1–2	119 311 (25.7)	224 (30.4)	5653 (24.6)	99 (22.9)
3–4	46 688 (10.1)	112 (15.2)	3424 (14.9)	48 (11.1)
>5	56 352 (12.1)	169 (22.9)	7017 (30.6)	138 (31.9)
Any inpatient visit in 2019	33 134 (7.1)	64 (8.7)	5359 (23.4)	65 (15.0)
Comorbidities				
Asthma	32 348 (7.0)	119 (16.1)	2584 (11.3)	66 (15.2)
Cancer	5572 (1.2)	8 (1.1)	1771 (7.7)	21 (4.8)
Cardiac or cerebrovascular disease	16 738 (3.6)	21 (2.8)	6785 (29.6)	61 (14.1)
Chronic kidney failure	4015 (0.9)	2 (0.3)	2315 (10.1)	18 (4.2)
Chronic liver disease	2655 (0.6)	2 (0.3)	544 (2.4)	6 (1.4)
Chronic lung disease	4714 (1.0)	6 (0.8)	2405 (10.5)	16 (3.7)
Diabetes	17 405 (3.8)	22 (3.0)	5015 (21.9)	86 (19.9)
Hypertension	46 258 (10.0)	69 (9.4)	10 740 (46.8)	167 (38.6)
Immunocompromised state	14 442 (3.1)	31 (4.2)	2678 (11.7)	43 (9.9)
Mental health disorder	120 140 (25.9)	376 (51.0)	6088 (26.5)	125 (28.9)
Neurological disorder	7211 (1.6)	5 (0.7)	1830 (8.0)	3 (0.7)
Obesity	23 053 (5.0)	39 (5.3)	2493 (10.9)	82 (18.9)
Number of symptoms in WHO PCC definition in 2019				
0	301 616 (65.0)	321 (43.6)	11 249 (49.0)	197 (45.5)
1	98 143 (21.2)	181 (24.6)	5785 (25.2)	120 (27.7)
2	39 839 (8.6)	122 (16.6)	3113 (13.6)	58 (13.4)
3	15 198 (3.3)	64 (8.7)	1523 (6.6)	38 (8.8)
>4	9141 (2.0)	49 (6.6)	1271 (5.5)	20 (4.6)
COVID-19 vaccination status				
Unvaccinated	277 103 (59.7)	589 (79.9)	16 526 (72.0)	425 (98.2)
1 dose	10 560 (2.3)	16 (2.2)	440 (1.9)	1 (0.2)
2 doses	142 492 (30.7)	110 (14.9)	1779 (7.8)	3 (0.7)
3 doses	31 063 (6.7)	21 (2.8)	2318 (10.1)	4 (0.9)
4 doses	2317 (0.5)	1 (0.1)	1575 (6.9)	0
>5 doses	402 (0.1)	0	303 (1.3)	0
SARS-CoV-2 variant period				
Wild type	165 710 (35.7)	422 (57.3)	10 826 (47.2)	279 (64.4)
Alpha	79 010 (17.0)	133 (18.0)	4180 (18.2)	132 (30.5)
Delta	37 605 (8.1)	53 (7.2)	1391 (6.1)	14 (3.2)
Omicron	181 612 (39.1)	129 (17.5)	6544 (28.5)	8 (1.8)
ICU admitted with COVID-19	NA	NA	1351 (5.9)	250 (57.7)
Number of visits at PCC clinic	NA	7.0 (4.0, 13.0)	NA	3.0 (1.0, 7.0)
Reason for end of follow-up				
Administrative	441 702 (95.2)	726 (98.5)	19 613 (85.5)	425 (98.2)
Death	4851 (1.0)	1 (0.1)	2901 (12.6)	4 (0.9)
Moving out of Stockholm County	17 384 (3.7)	10 (1.4)	427 (1.9)	4 (0.9)

Data are presented as numbers and percentages or medians and interquartile intervals.

* Data were missing for 1287 participants who were excluded from these analyses.

† Data were missing for 10 469 participants who were excluded from these analyses.

‡ Data were missing for 1338 participants who were excluded from these analyses.

ICU, intensive care unit; PCC, post-COVID-19 condition; WHO, World Health Organization.

Characteristics of the cohort with new-onset symptoms in primary care by severity of infection and PCC clinic attendance are presented in table 2. As for the overall cohort, among non-hospitalised participants, education level was higher in participants who attended a PCC clinic compared with those who did not attend, whereas no such difference in education level was observed among hospitalised participants.

**Table 2:** Characteristics of participants with ≥1 new-onset PCC symptom diagnosis codes registered in primary care by COVID-19 severity and PCC clinic attendance

	Not hospitalised (n=75 878)		Hospitalised (n=6190)	
	No visit (n=75 394)	>1 visit (n=484)	No visit (n=5993)	>1 visit (n=197)
Female sex	50 088 (66.4)	369 (76.2)	3064 (51.1)	86 (43.7)
Age, years	43.0 (33.0, 54.0)	45.5 (38.0, 51.2)	67.0 (54.0, 80.0)	56.0 (47.0, 62.0)
Age category, years				
18–29	13 058 (17.3)	31 (6.4)	148 (2.5)	5 (2.5)
30–39	17 557 (23.3)	104 (21.5)	355 (5.9)	13 (6.6)
40–49	18 168 (24.1)	199 (41.1)	586 (9.8)	43 (21.8)
50–59	14 889 (19.7)	117 (24.2)	1007 (16.8)	70 (35.5)
60–69	7050 (9.4)	32 (6.6)	1146 (19.1)	45 (22.8)
70–79	3045 (4.0)	1 (0.2)	1239 (20.7)	19 (9.6)
>80	1627 (2.2)	0	1512 (25.2)	2 (1.0)
Born in Sweden*	50 618 (67.2)	376 (77.7)	3383 (56.5)	105 (53.3)
Education level†				
Primary	9664 (13.0)	23 (4.8)	1421 (24.5)	41 (21.1)
Secondary	27 950 (37.7)	159 (33.0)	2429 (41.8)	87 (44.8)
Tertiary	36 450 (49.2)	300 (62.2)	1960 (33.7)	66 (34.0)
Age-standardised income quartile‡				
First	15 836 (21.0)	99 (20.5)	2044 (34.1)	54 (27.4)
Second	22 667 (30.1)	153 (31.6)	1713 (28.6)	64 (32.5)
Third	20 614 (27.4)	145 (30.0)	1281 (21.4)	56 (28.4)
Fourth	16 202 (21.5)	87 (18.0)	954 (15.9)	23 (11.7)
Number of sick days in 2019‡				
0	65 359 (86.8)	373 (77.1)	5373 (89.7)	149 (75.6)
1–30	4334 (5.8)	43 (8.9)	219 (3.7)	18 (9.1)
>31	5626 (7.5)	68 (14.0)	400 (6.7)	30 (15.2)
Number of primary care visits in 2019				
0	20 280 (26.9)	122 (25.2)	863 (14.4)	36 (18.3)
1–2	28 503 (37.8)	184 (38.0)	1844 (30.8)	62 (31.5)
3–4	13 925 (18.5)	100 (20.7)	1306 (21.8)	48 (24.4)
>5	12 686 (16.8)	78 (16.1)	1980 (33.0)	51 (25.9)
Number of outpatient specialist care visits in 2019				
0	30 718 (40.7)	149 (30.8)	1480 (24.7)	67 (34.0)
1–2	21 433 (28.4)	164 (33.9)	1503 (25.1)	50 (25.4)
3–4	9748 (12.9)	75 (15.5)	966 (16.1)	19 (9.6)
>5	13 495 (17.9)	96 (19.8)	2044 (34.1)	61 (31.0)
Any inpatient visit in 2019	6525 (8.7)	40 (8.3)	1393 (23.2)	27 (13.7)
Comorbidities				
Asthma	7364 (9.8)	84 (17.4)	855 (14.3)	36 (18.3)
Cancer	985 (1.3)	4 (0.8)	412 (6.9)	9 (4.6)
Cardiac or cerebrovascular disease	3501 (4.6)	11 (2.3)	1797 (30.0)	21 (10.7)
Chronic kidney failure	730 (1.0)	1 (0.2)	586 (9.8)	2 (1.0)
Chronic liver disease	543 (0.7)	1 (0.2)	133 (2.2)	1 (0.5)
Chronic lung disease	1120 (1.5)	4 (0.8)	692 (11.5)	6 (3.0)
Diabetes	3549 (4.7)	11 (2.3)	1324 (22.1)	27 (13.7)
Hypertension	9866 (13.1)	40 (8.3)	2970 (49.6)	70 (35.5)
Immunocompromised state	2777 (3.7)	20 (4.1)	713 (11.9)	17 (8.6)
Mental health disorder	31 698 (42.0)	259 (53.5)	2191 (36.6)	56 (28.4)
Neurological disorder	1045 (1.4)	3 (0.6)	394 (6.6)	1 (0.5)
Obesity	5922 (7.9)	23 (4.8)	824 (13.7)	38 (19.3)
Number of symptoms in WHO PCC definition in 2019				
0	39 222 (52.0)	220 (45.5)	2441 (40.7)	79 (40.1)
1	19 523 (25.9)	114 (23.6)	1620 (27.0)	63 (32.0)
2	9512 (12.6)	75 (15.5)	963 (16.1)	26 (13.2)
3	4197 (5.6)	43 (8.9)	523 (8.7)	18 (9.1)
>4	2940 (3.9)	32 (6.6)	446 (7.4)	11 (5.6)
COVID-19 vaccination status				
Unvaccinated	44 048 (58.4)	383 (79.1)	4320 (72.1)	191 (97.0)
1 dose	1881 (2.5)	12 (2.5)	111 (1.9)	0
2 doses	23 095 (30.6)	74 (15.3)	431 (7.2)	2 (1.0)
3 doses	5851 (7.8)	14 (2.9)	630 (10.5)	4 (2.0)
4 doses	446 (0.6)	1 (0.2)	426 (7.1)	0
>5 doses	73 (0.1)	0	75 (1.3)	0
SARS-CoV-2 variant period				
Wild type	27 133 (36.0)	279 (57.6)	2863 (47.8)	135 (68.5)
Alpha	11 449 (15.2)	87 (18.0)	1072 (17.9)	48 (24.4)
Delta	6512 (8.6)	33 (6.8)	400 (6.7)	9 (4.6)
Omicron	30 300 (40.2)	85 (17.6)	1658 (27.7)	5 (2.5)
Admitted to ICU with COVID-19	NA	NA	448 (7.5)	113 (57.4)
Number of visits at PCC clinic	NA	7.0 (4.0, 15.0)	NA	3.0 (2.0, 7.0)
Reason for end of follow-up				
Administrative	72 118 (95.7)	476 (98.3)	5251 (87.6)	193 (98.0)
Death	740 (1.0)	0	642 (10.7)	1 (0.5)
Moving out of Stockholm County	2536 (3.4)	8 (1.7)	100 (1.7)	3 (1.5)

Data are presented as numbers and percentages or medians and interquartile intervals.

* Data were missing for 73 participants who were excluded from these analyses.

† Data were missing for 1518 participants who were excluded from these analyses.

‡ Data were missing for 76 participants who were excluded from these analyses.

ICU, intensive care unit; PCC, post-COVID-19 condition; WHO, World Health Organization.

### Factors associated with post-COVID-19 condition (PCC) clinic attendance

**Figure 2:**
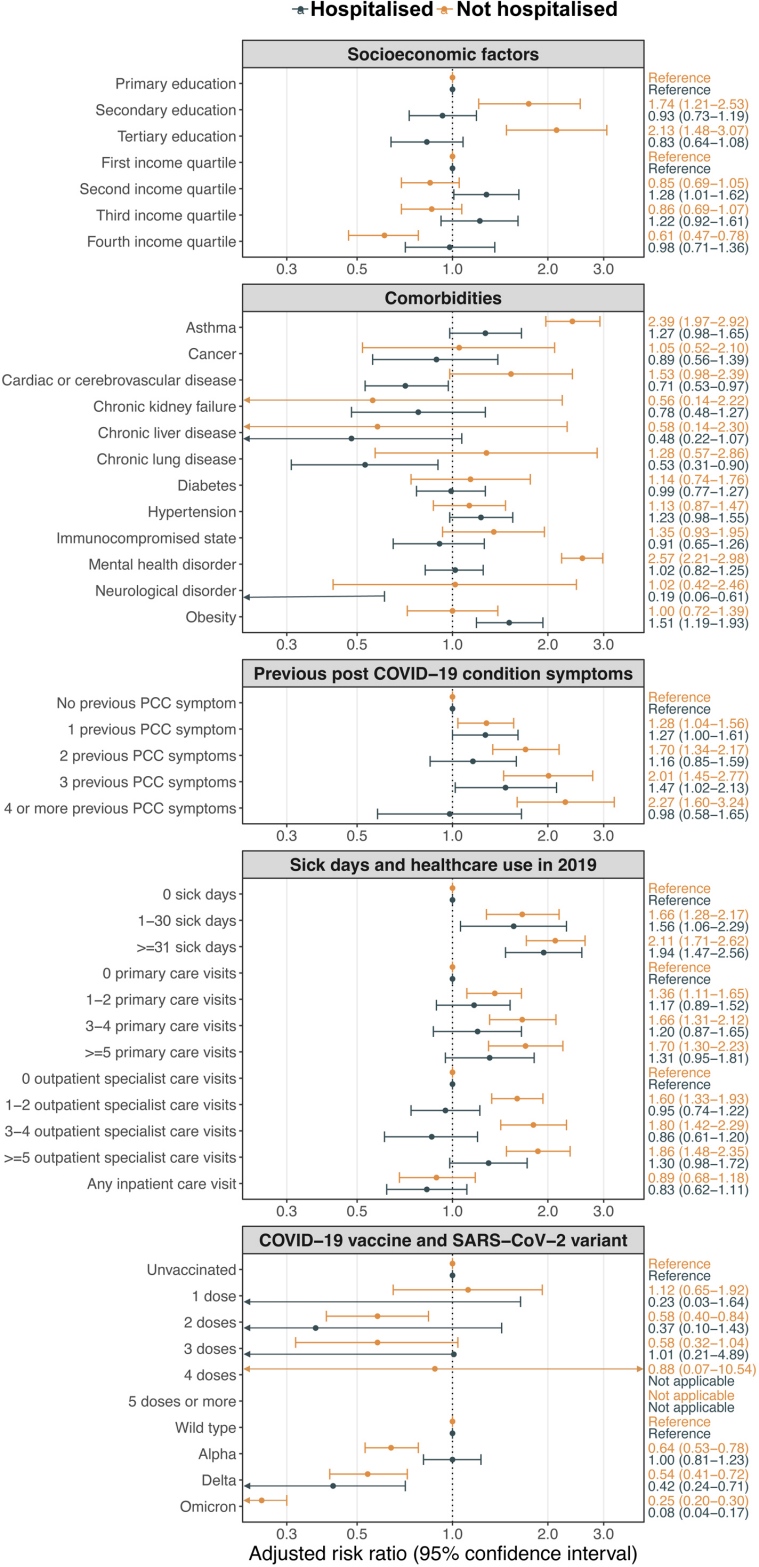
Forest plot of baseline characteristics and adjusted risk ratios of PCC clinic attendance. Confounders for each model are described in online supplemental table S3. None of the hospitalised participants who had received ≥4 doses of COVID-19 vaccine attended a PCC clinic. None of the non-hospitalised participants who had received >5 doses of COVID-19 vaccine attended a PCC clinic. PCC, post-COVID-19 condition.

The unadjusted RR (95% CI) for attending a PCC clinic and being female compared with male was 2.40 (2.03–2.83) among non-hospitalised and 0.81 (0.67–0.98) among hospitalised participants (online supplemental figure S2). Compared with non-hospitalised participants aged 18 to 29 years, non-hospitalised participants aged 30 to 69 years had an increased risk of attending a PCC clinic. The five strongest adjusted associations were all among non-hospitalised participants, being mental health disorder (adjusted RR 2.57, 95% CI 2.21 to 2.98), asthma (2.39, 1.97–2.92), >4 symptoms that could indicate PCC in primary care in 2019 (2.27, 1.60–3.24), tertiary education (2.13, 1.48–3.07) and >31 sick days in 2019 (2.11, 1.71–2.62) (figure 2). Among hospitalised participants, the strongest associations were >31 sick days in 2019 (adjusted RR 1.94, 95% CI 1.47 to 2.56), 1–30 sick days in 2019 (1.56, 1.06–2.29) and obesity (1.51, 1.19–1.93). Adjusted results were almost identical when using Fine-Gray models instead of modified Poisson regression models (online supplemental figures S3 and S4). The above results were similar in the sensitivity analysis also including visits to the PCC clinic at KUHS (online supplemental table S1).

When restricting the analyses to 82 068 participants who had one or more symptom diagnosis codes that could indicate PCC in primary care, many of the associations observed in the main analyses were weaker or not present (online supplemental figures S5 and S6). Higher education level, asthma and mental health disorder were still associated with PCC clinic attendance among non-hospitalised participants. A total of 3954 participants (2920 not hospitalised and 1034 hospitalised) received a PCC diagnosis in primary care but did not attend a PCC clinic (online supplemental table S5). Among the non-hospitalised participants, 588 (79.8%) of those that attended a PCC clinic versus 2005 (68.7%) of those that received a PCC diagnosis in primary care only were born in Sweden. No such difference was observed among hospitalised participants (51.7% and 55.5%, respectively).

### Diagnosis codes registered at post-COVID-19 condition (PCC) clinics

**Figure 3:**
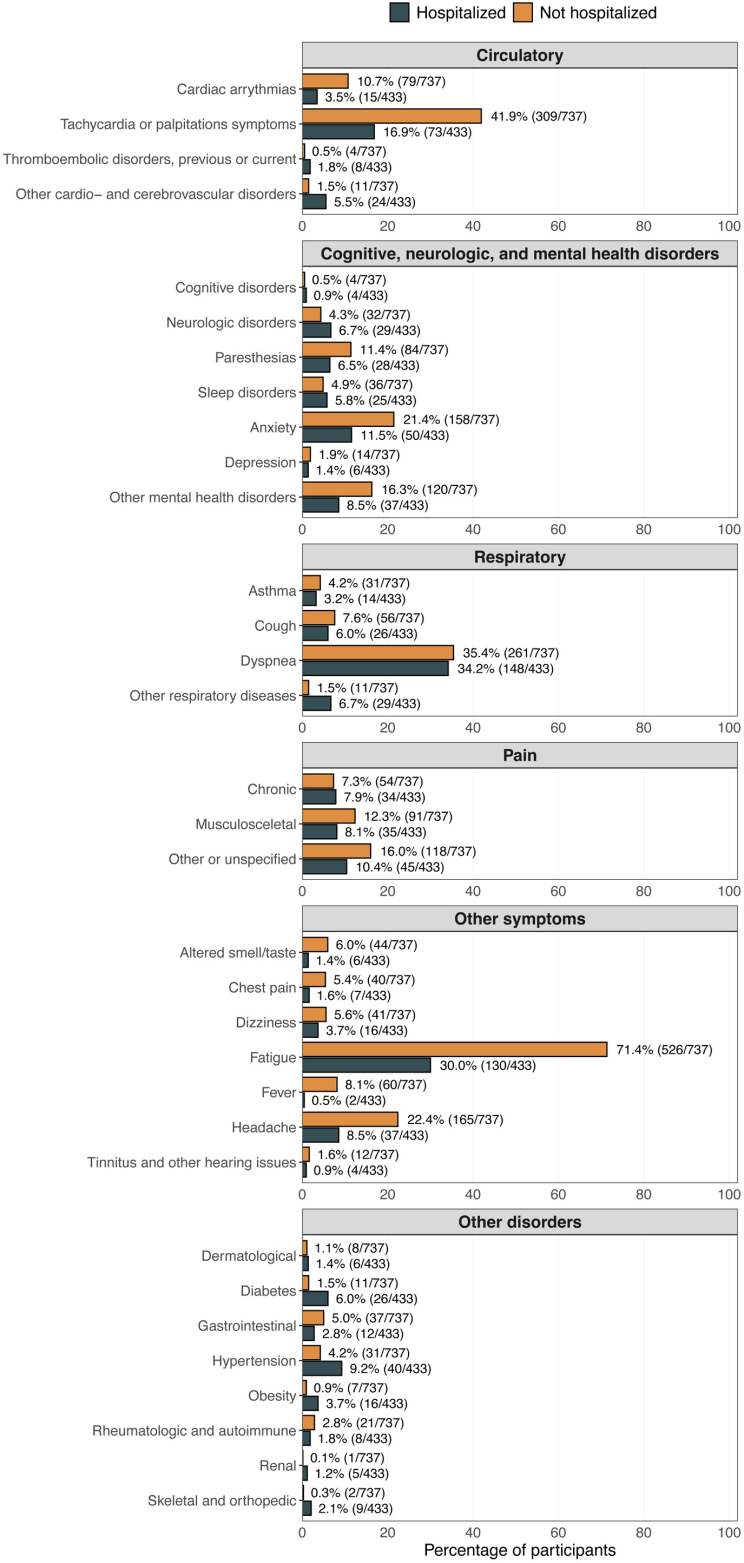
Symptoms and diagnoses registered at PCC clinics. Classification of all diagnosis codes is presented in online supplemental table S2. PCC, post-COVID-19 condition.

Of the 1170 participants that attended a PCC clinic, 962 (82.2%) had a PCC diagnosis (U09.9) registered during a PCC clinic visit, being 689 (93.5%) among non-hospitalised participants and 273 (63.0%) among hospitalised participants. Besides the diagnoses of COVID-19 and PCC, fatigue was the most common symptom among participants who were not hospitalised (n=526, 71.4%), whereas dyspnoea was the most common among participants who were hospitalised (n=148, 34.2%) (figure 3).

## Discussion

In this population-based cohort study of 488 048 participants with a verified SARS-CoV-2 infection, 0.2% of non-hospitalised and 1.9% of hospitalised participants attended a PCC clinic. We observed marked differences in factors associated with PCC clinic attendance between these groups. Fatigue was the most common symptom registered among non-hospitalised participants, whereas dyspnoea dominated among those hospitalised. A total of 16.3% of the non-hospitalised participants and 26.5% of the hospitalised participants were presented with new-onset symptoms potentially indicating PCC in primary care.

To our knowledge, no previous population-based study has examined determinants of attendance at PCC clinics. Our results help characterise how this novel care setting has been utilised and reveal potential disparities in care access. A US study of 252 PCC clinic attendees reported a median age of 44.5 years, with 63.5% of patients being female, and the most common comorbidities included mental health conditions, hypertension and asthma.^10^ These findings closely mirror ours, where the median age was 49 years, 61.6% were female and similar comorbidity burdens were observed. The US study also noted an overrepresentation of patients with private insurance and college education, which the authors suggested may reflect a more healthcare-seeking behaviour and higher capacity to obtain care.

Several studies exclusively follow PCC clinic patients who have been hospitalised, and it is important to understand if patients attending PCC clinics differ from others with established SARS-CoV-2 exposure, which may affect the external validity of these studies. This is exemplified by a living systematic review of PCC characteristics, where 78% of included participants were hospitalised during the acute SARS-CoV-2 infection.^11^

We showed that higher education was associated with PCC clinic attendance. A history of healthcare visits both in primary care and specialised outpatient care was overrepresented among non-hospitalised individuals coming to PCC. Since less access to PCC clinics would be expected for individuals with less severe infections, a greater health literacy and awareness of care pathways (including self-referrals and previous healthcare experiences) might have facilitated PCC clinic attendance. Thus, more vulnerable or disadvantaged parts of the community may not have received adequate care. Furthermore, patients and caregivers may find it difficult to distinguish pre-existing symptoms from new ones. Importantly, access to PCC services may be influenced by systemic barriers such as referral practices, language and socioeconomic constraints. The observed association between higher education and Swedish birth with PCC clinic attendance suggests inequities in access to care. These disparities may reflect differences in health literacy, capacity to navigate the healthcare system or implicit bias in referral decisions. Clinic attendance likely reflects not only clinical need but also awareness and availability. Media attention, public concern and clinic capacity during early waves may have affected PCC identification. Such contextual factors should be considered when interpreting absolute attendance rates.

In our study, the symptom presentation differed between non-hospitalised and hospitalised participants, indicating heterogeneity in the clinical presentation of PCC. Similar to our findings, a 2023 meta-analysis found that 34.8% of non-hospitalised participants had fatigue.^12^ The study also concluded that the definition of PCC subtypes is unclear, which may hamper effective treatment and management strategies.

A total of 82 068 participants were included in the analysis of participants who had a symptom diagnosis code that could indicate PCC in primary care. While it is unlikely that all participants had a clinical presentation that could be attributed to PCC, it highlights a challenge for primary care physicians to adequately identify patients with PCC who could benefit from referral to specialised care. The epidemiological research on PCC has suffered from an overly broad case definition and absence of control groups.^13^ This impairs the use of existing scientific literature to establish evidence-based recommendations for PCC. A recent study of patients attending the emergency department found that over one-third of patients who were positive for SARS-CoV-2 met the WHO PCC criteria 3 months after the emergency department visit.^14^ However, a fifth of those that were negative for SARS-CoV-2 also reported symptoms consistent with PCC, which highlighted the low specificity of the clinical case definition.

An important strength of this study is the cross-linking of several population-based registers, which enabled us to not only identify visits at PCC clinics, but also visits in primary care where a PCC diagnosis was registered. Furthermore, by having access to not only all positive SARS-CoV-2 PCR tests, but also positive SARS-CoV-2 serology tests during 2020, we could include around 50 000 participants who had an infection during 2020, primarily non-hospitalised participants. Importantly, we were able to distinguish between participants who were not hospitalised versus hospitalised during the acute SARS-CoV-2 infection, which enabled us to demonstrate large differences in both factors influencing PCC clinic attendance and symptoms reported during those visits. Importantly, the internal validity is high, since we had access to all PCC clinic visits in Stockholm County. Furthermore, we had access to a wide range of important epidemiological and clinical data, including but not limited to education and income status, comorbidities, previous PCC symptoms, sick days and healthcare use before the pandemic, and COVID-19 vaccinations.

The generalisability of these findings is difficult to assess due to a lack of comparable studies. This study was set in the capital region of a high-income country with publicly funded healthcare services, thus reducing selection bias. Our results might, however, not be generalisable to settings with different healthcare funding systems. Furthermore, due to the overlap between PCC symptoms and those of other conditions, the use of ICD-coded symptoms and clinic attendance may have introduced outcome misclassification. Importantly, symptom codes registered in primary care or during PCC-related visits may not necessarily reflect PCC. This may result in both false positive and false negative classifications, particularly in the absence of standardised clinical adjudication. Future studies incorporating objective functional assessments and standardised diagnostic criteria are needed to improve PCC case ascertainment. Importantly, we did not collect data on treatments (eg, rehabilitation, medication), nor symptom severity or duration. These factors are critical to understanding recovery trajectories and treatment effects. Inclusion of such data should be prioritised in future longitudinal studies. Granular data on the severity of the infection among non-hospitalised participants were not available. Finally, our results were based on first SARS-CoV-2 infections, and the effect of SARS-CoV-2 reinfections was not considered.

In conclusion, attending a PCC clinic after a SARS-CoV-2 infection was more frequent among hospitalised participants, but non-hospitalised participants accounted for most visits. Pre-existing mental health disorder and PCC symptoms the year before the pandemic were strongly associated with PCC clinic attendance in those that were not hospitalised. The findings suggest that distinguishing PCC from conditions with overlapping symptoms, and thereby determining the appropriate level of care, may be challenging, with risk of displacement effects and inappropriate care. Significant socioeconomic disparities in PCC clinic attendance were also identified. More specific PCC criteria and targeted efforts may increase equitable and clinically relevant access to PCC services. Future research should explore stratified care models and targeted interventions to improve equity and guide appropriate service provision.

## Supplementary material

10.1136/bmjopen-2024-098344 online supplemental file 1

## Data Availability

No data are available.
