# Supplementary material for: Determinants of post COVID-19 clinic attendance among SARS-CoV-2-infected individuals in Stockholm, Sweden: a population-based cohort study
Source: BMJ Open. 2025 Jun 17;15(6):e098344. doi: 10.1136/bmjopen-2024-098344 (PMC12182103; doi:10.1136/bmjopen-2024-098344)
Supplement: online supplemental file 1 [file bmjopen-15-6-s001.docx]

Supplementary material for

**Determinants of post COVID-19 clinic attendance among SARS-CoV-2 infected individuals in Stockholm, Sweden: A population-based cohort study**

**Authors:** Pontus Hedberg, Peder af Geijerstam, John Karlsson Valik, Christer Almgren-Lidman, Anders Ternhag, Pontus Nauclér

Correspondence to pontus.hedberg@ki.se

**Table of contents**

| **Content** | **Page** |
| --- | --- |
| Table S1. Study variable definitions, sources and missingness | 3-7 |
| Table S2. Classification of diagnosis codes registered during PCC clinic visits | 8-9 |
| Table S3. Confounders in the adjusted models | 10 |
| Figure S1. PCC clinic visits over time among non-hospitalized and hospitalized participants | 11 |
| Table S4. Adjusted risk ratios vs adjusted subdistribution hazard ratios among hospitalized participants | 12-13 |
| Figure S2. Forest plot of baseline characteristics and unadjusted risk ratios of PCC clinic attendance | 14 |
| Figure S3. Forest plot of baseline characteristics and adjusted risk ratios of PCC clinic attendance in the main analysis vs the sensitivity analysis among non-hospitalized participants | 15 |
| Figure S4. Forest plot of baseline characteristics and adjusted risk ratios of PCC clinic attendance in the main analysis vs the sensitivity analysis among hospitalized participants | 16 |
| Figure S5. Forest plot of baseline characteristics and unadjusted risk ratios of PCC clinic attendance among participants with new-onset symptoms that could indicate PCC in primary care | 17 |
| Figure S6. Forest plot of baseline characteristics and adjusted risk ratios of PCC clinic attendance among participants with new-onset symptoms that could indicate PCC in primary care | 18 |
| Table S5. Comparison of participants with a PCC diagnosis registered in primary care and participants attending a PCC clinic | 19-21 |

**Table S1. Study variable definitions, sources and missingness**

| **Variable** | **Data sources** | **Missing data** | **Definition** | **Time period** | **Possible values** |
| --- | --- | --- | --- | --- | --- |
| **Outcome variables** |  |  |  |  |  |
| PCC clinic visit | Intelligence, VAL, SmiNet | No | No PCC clinic visit: No visit at any of the two PCC clinics  Any PCC clinic visit: One or more visits at any of the two PCC clinics | From 90 days after the first positive SARS-CoV-2 PCR or serology test until end of follow-up (date of death, moving out of Stockholm County, or November 30, 2023) | Yes, No |
| Number of visits at PCC clinic | Intelligence, VAL, SmiNet | No | Number of calendar dates with a visit at any of the two PCC clinics | From 90 days after the first positive SARS-CoV-2 PCR or serology test until end of follow-up (date of death, moving out of Stockholm County, or November 30, 2023) | 0-67 |
| Symptom/Disease group at PCC visit | Intelligence, VAL, SmiNet | No | Symptoms and diseases were classified in accordance with the definitions presented in Table S2. | PCC visits from 90 days after the first positive SARS-CoV-2 PCR or serology test until end of follow-up (date of death, moving out of Stockholm County, or November 30, 2023) | See Table S2 |
| PCC diagnosis in primary care | VAL, SmiNet | No | No PCC diagnosis: No PCC diagnosis in primary care  Any PCC diagnosis: One or more PCC diagnoses in primary care | From 90 days after the first positive SARS-CoV-2 PCR or serology test until end of follow-up (date of death, moving out of Stockholm County, or November 30, 2023) | Yes, No |
| PCC clinic visit, including Karolinska University Hospital Solna | Intelligence, VAL, SmiNet | No | No PCC clinic visit: No visit at any of the two PCC clinics or PCC clinic at Karolinska University Hospital Solna  Any PCC clinic visit: One or more visits at any of the two PCC clinics or PCC clinic at Karolinska University Hospital Solna | From 90 days after the first positive SARS-CoV-2 PCR or serology test until end of follow-up (date of death, moving out of Stockholm County, or November 30, 2023). Since this clinic did not have its own administrative code, we identified these visits by including all COVID-19 follow-up visits at the pulmonary and infectious diseases clinics where a 6-Minute Walk Test was performed. This examination was widely used at this clinic from April 2020. | Yes, No |
| **Baseline characteristics** |  |  |  |  |  |
| Sex | VAL | No | Biological sex | Birth | Male, Female |
| Age | VAL, SmiNet | No | Age at the date of the first positive SARS-CoV-2 PCR test | - | 18-107 years |
| Age category | VAL, SmiNet | No | Age category at the date of the first positive SARS-CoV-2 PCR test | - | 18-29, 30-39, 40-49, 50-59, 60-69, 70-79, 80 years or older |
| Born in Sweden | Statistics Sweden | Yes, for 1287 individuals | Born in Sweden | Birth | Yes, No, Missing |
| Education level | Statistics Sweden | Yes, for 10 469 individuals | Education level in 2019 | End of 2019 | Primary, Secondary, Tertiary, Missing |
| Age-standardized income quartile | Statistics Sweden | Yes, for 1338 individuals | The birthyear stratified yearly disposable income quartile in 2019 | 2019 | Quartile 1, Quartile 2, Quartile 3, Quartile 4, Missing |
| Number of sick days in 2019 | Statistics Sweden | Yes, for 1338 individuals | Number of sick days in 2019 | 2019 | 0, 1-30, >31, Missing |
| Primary care visits in 2019 | VAL | No | Number of primary care visits to a medical doctor in 2019 | January 1, 2019, until December 31, 2019 | 0, 1-2, 3-4, >5 |
| Outpatient specialist care visits in 2019 | VAL | No | Number of outpatient specialist care visits to a medical doctor in 2019 | January 1, 2019, until December 31, 2019 | 0, 1-2, 3-4, >5 |
| Any inpatient visit in 2019 | VAL | No | Any inpatient visit in 2019 | January 1, 2019, until December 31, 2019 | Yes, No |
| Asthma | VAL, SmiNet | No | ICD-10: J45.X, J46.X | From 5 years to 14 days before the first positive SARS-CoV-2 PCR test | Yes, No |
| Cancer | VAL, SmiNet | No | ICD-10: All codes from C00.X to C97.X besides C44.X, Z51.0, Z51.1  KVÅ: DT107, DT108, DT112, DT116, DT135, DV070, DV071, DV134 | From 1 year to 14 days before the first positive SARS-CoV-2 PCR test  From 1 year to 14 days before the first positive SARS-CoV-2 PCR test | Yes, No |
| Cardiac or cerebrovascular disease | VAL, SmiNet | No | ICD-10: I20.X, I21.X, I22.X, I23.X, I24.X, I25.X, I26.X, I27.X, I42.X, I48.X, I50.X, I61.X, I63.X, I64.X | From 5 years to 14 days before the first positive SARS-CoV-2 PCR test  From 5 years to 14 days before the first positive SARS-CoV-2 PCR test | Yes, No |
| Chronic kidney failure | VAL, SmiNet | No | ICD-10: N18.X  ICD-10: Z49.1, Z49.2 (should be registered at least 12 times during the time period)  ICD-10: Z99.2  KVÅ: DR016, DR024 (should be registered at least 12 times during the time period) | From 5 years to 14 days before the first positive SARS-CoV-2 PCR test  From 1 year to 14 days before the first positive SARS-CoV-2 PCR test  From 1 year to 14 days before the first positive SARS-CoV-2 PCR test  From 1 year to 14 days before the first positive SARS-CoV-2 PCR test | Yes, No |
| Chronic liver disease | VAL, SmiNet | No | ICD-10: B18.X, K70.X, K71.7, K72.X, K74.6, K75.X | From 5 years to 14 days before the first positive SARS-CoV-2 PCR test | Yes, No |
| Chronic lung disease | VAL, SmiNet | No | ICD-10: D86.0, D86.2, E84.X, J43.X, J44.X, J46.9, J47.X, J70.3, J84.X, J98.2 | From 5 years to 14 days before the first positive SARS-CoV-2 PCR test | Yes, No |
| Diabetes | VAL, SmiNet | No | ICD-10: E10.X, E11.X | From 5 years to 14 days before the first positive SARS-CoV-2 PCR test | Yes, No |
| Hypertension | VAL, SmiNet | No | ICD-10: I10.X, I11.X, I12.X, I13.X, 15.X (should be registered at least twice during the time period) | From 5 years to 14 days before the first positive SARS-CoV-2 PCR test | Yes, No |
| Immunocompromised state | VAL, SmiNet | No | ATC: H02AB.X (should be registered at least twice during the period)  ATC: L01.X  ATC: L04.X  ICD-10: B20.X, B21.X, B22.X, B23.X, B24.X, D57.0, D57.1, D80.X, D81.X, D82.X, D83.X, D84.X, Z94.0, Z94.1, Z94.2, Z94.3, Z94.4, Z94.8  KVÅ: DR04.1, DR04.2, DR04.4, DR04.6, DR04.7  KVÅ: H02AB.X (should be registered at least twice during the period)  KVÅ: L01.X  KVÅ: L04.X | From 6 months to 14 days before the first positive SARS-CoV-2 PCR test  From 1 year to 14 days before the first positive SARS-CoV-2 PCR test  From 6 months to 14 days before the first positive SARS-CoV-2 PCR test  From any time until 14 days before the first positive SARS-CoV-2 PCR test  From 3 years to 14 days before the first positive SARS-CoV-2 PCR test  From 6 months to 14 days before the first positive SARS-CoV-2 PCR test  From 1 year to 14 days before the first positive SARS-CoV-2 PCR test  From 6 months to 14 days before the first positive SARS-CoV-2 PCR test | Yes, No |
| Mental health disorder | VAL, SmiNet | No | ICD-10: F20.X-F29.X, F30.X-F39.X, F40.X-F48.X | From 5 years to 14 days before the first positive SARS-CoV-2 PCR test | Yes, No |
| Neurological disorder | VAL, SmiNet | No | ICD-10: F00.X, F01.X, F02.X, F03.X, G10.X, G12.2, G20.X, G30.X, G35.X, G70.X, G71.X, G80.X | From 5 years to 14 days before the first positive SARS-CoV-2 PCR test | Yes, No |
| Obesity | VAL, SmiNet | No | ICD-10: E66.X | From 5 years to 14 days before the first positive SARS-CoV-2 PCR test | Yes, No |
| Number of symptoms in WHO PCC definition in 2019 | VAL | No | ICD-10 codes: Abdominal pain: R10.X  Altered smell/taste: R43.X  Anxiety: F40.X-F48.X  Chest pain: R07.1, R07.2, R07.3  Cough: R05.X  Depression: F30.X-F39.X  Dizziness: R42.X  Dyspnoea: R06.0 Fatigue: G93.3, R53.X  Fever: R50.9 Gastrointestinal issues (diarrhoea, constipation, acid reflux): K51.X, K58.X, K59.0, K59.1, K59.2, R11.X, R12.X, R19.4  Headache: G43.X, G44.X, R51.X  Joint pain: M25.5  Myalgia: M79.1, M79.2, M79.6, M79.9, R25.2  Neuralgia: G50.0, M79.2, R52.2  Paraesthesia: R20.2  Sleep disorder: G47.X  Tachycardia/palpitations: I47.X, I49.5, R00.0, R00.2  Tinnitus and other hearing issues: H93.1, H93.2 | January 1, 2019, until December 31, 2019 | 0, 1, 2, 3, >4 |
| COVID-19 vaccination status before infection | NVR, SmiNet | No | Number of COVID-19 vaccine doses received | From December 27, 2020, to 14 days before the first positive SARS-CoV-2 PCR test | Unvaccinated, 1 dose, 2 doses, 3 doses, 4 doses, >5 doses |
| SARS-CoV-2 variant period | SmiNet | No | Wild type: Positive SARS-CoV-2 PCR test any time from October 1, 2020, to February 14, 2021  Alpha: Positive SARS-CoV-2 PCR test any time from February 15, 2021, to June 27, 2021  Delta: Positive SARS-CoV-2 PCR test any time from June 28, 2021, to December 26, 2021  Omicron: Positive SARS-CoV-2 PCR test any time from December 27, 2021, to November 30, 2022 | From October 1, 2020, to November 30, 2022 | Wild type, Alpha, Delta, Omicron |
| COVID-19 severity | VAL, SIR | No | Hospitalized: A hospital admission with a first positive SARS-CoV-2 PCR test and a U07.1 or U07.2 ICD-10 code as main or secondary diagnosis at discharge  ICU-treated: Hospitalized in accordance with the definition above plus admission to the ICU any time during this hospitalization.  Not hospitalized: None of the above | Any time from 14 days before admission up until date of discharge | Not hospitalized, hospitalized, ICU-treated |
| Reason for end of follow-up | VAL | No | Reason for end of follow-up being death, moving out of Stockholm County, or administrative, whichever occurred first. | Date of death, date of moving out of Stockholm County, or November 30, 2023, whichever occurred first. | Administrative, Death, Moving out of Stockholm County |

**Abbreviations:** ATC=Anatomical Therapeutic Chemical, COVID-19=Coronavirus disease 2019, ICD-10=International Classification of Diseases 10^th^ revision; ICU=Intensive care unit, KVÅ=Klassifikation av vårdåtgärder (Swedish for classification of healthcare procedures), PCC=Post COVID-19 condition, SARS-CoV-2=Severe acute respiratory syndrome coronavirus 2, SIR=Swedish Intensive Care Registry, VAL= Stockholm regional healthcare data warehouse, WHO=World Health Organization

**Table S2. Classification of diagnosis codes registered during PCC clinic visits**

| **Symptom/Disease group** | **ICD-10 diagnosis codes** |
| --- | --- |
| COVID-19, current/previous | U07.1, U07.2, U08.9 |
| PCC diagnosis | U09.9 |
| Cardiac arrythmias | I48.0, I48.2, I48.9, I49.5, I49.5B, I49.8, I49.8E, I49.9, R00.1 |
| Tachycardia or palpitations symptoms | I47.1, R00.0, R00.2 |
| Thromboembolic disorders, previous or current | I26.9, Z86.7A, Z86.7B |
| Other cardio- and cerebrovascular disorders | I07.1, I20.1, I20.8, I20.9, I21.9, I25.1, I25.2, I25.9, I27.9, I35.0, I35.1, I40.8, I42.0, I42.2, I42.9, I50.1, I50.9, I51.4, I61.2, I61.6, I61.9, I63.9, I69.1, I71.2, I72.2A, I80.1, Z86.6A, Z86.7, Z95.0, Z95.1, Z95.2, Z95.3, Z95.8 |
| Cognitive disorders | R41.0, R41.2, R41.3, R41.8A |
| Neurologic disorders | G09.9, G14.9, G20.9, G24.9, G25.0, G35.9, G37.9, G40.9, G54.5, G60.0, G61.0, G61.9, G62.8, G62.9, G64.9, G71.0, G71.8, G72.8, G72.9, G81.1, G81.9, G83.1, G83.8, G83.9, G90.0, G90.8, G90.9, G93.1, G93.2, Q00-Q99, R47.0A, R25.1 |
| Paraesthesia | R20.2, R20.8 |
| Sleep disorders | G47.3, G47.9 |
| Anxiety | F40-F48 |
| Depression | F31, F32, F33, F34 |
| Other mental health disorders | F06, F10-F19, F20-F29, F50-F59, F60-F69, F80-F89, F90-F98, R45.8 |
| Asthma | J45.0, J45.1, J45.8, J45.9, J46.9 |
| Cough | R05.9 |
| Dyspnoea | R06.0 |
| Other respiratory diseases | J00.9, J01.9, J12.8, J15.9, J30.1, J32.0, J32.9, J44.0, J44.1, J80.9C, J96.0, D86.0, J44.9, J47.9, J84.1, J96.1, J96.10, J96.9, J98.0, J98.8, J98.9 |
| Pain, chronic | R52.2, R52.2A, R52.2B, R52.2C |
| Pain, musculoskeletal | M25.5, M25.5B, M25.5F, M51.0J, M54.2, M54.4, M54.5, M54.6, M54.9, M79.1, M79.6, M79.6B, M79.6H, M79.7, M94.0, |
| Pain, other or unspecified | G50.0, M79.2, R52.9 |
| Altered smell/taste | R43.0, R43.1, R43.8 |
| Chest pain | R07.4 |
| Dizziness | H81.9, H82.9, R42.9 |
| Fatigue | G93.3, R53.9, Z73.0 |
| Fever | R50.9 |
| Headache | G43.1, G43.3, G43.9, G44.8, R51.9 |
| Tinnitus and other hearing issues | H90.3, H90.5, H91.9, H93.1, H93.2 |
| Dermatological disorders | L00-L08, L20-L30, L40-L45, L55-L59, L60-L75, L80-L99 |
| Diabetes | E10.3, E10.7, E10.9, E11.3, E11.4D, E11.7, E11.8, E11.9, E14.9 |
| Gastrointestinal disorders/symptoms | K21.9, K27.9, K29.7, K30.9, K50.9, K51.2, K52.8, K52.9, K58.1, K58.3, K58.8, K59.0, K59.1, K59.8, K75.4, K76.0, K83.0A, K90.0, R10.4X, R11.9, R11.9A, R13.9, R19.4 |
| Hypertension | I10.9 |
| Obesity | E66.0, E66.2, E66.9 |
| Rheumatologic and autoimmune disorders | E03.9, M05.9, M05.9L, M06.4, M06.9, M07.3X, M31.3, M32.1, M32.9, M35.0A, M35.2, M35.3 |
| Renal disorders | N18.3, N18.4, N18.5, N20.0, Z94.0 |
| Skeletal and orthopaedic disorders | M15.9, M17.0, M17.9, M21.3, M24.5H, M70.6, M75.1, M81.9 |
| Excluded from analyses | B02.3, B91.9, C79.7, C91.1, C91.4, C92.0, D35.2, D47.3, D50.9, D56.9, D58.0, D68.5C, D68.6A, E20-E35, E50-E64, E70-E90, G56.0, G56.2C, G57.1, G58.7, H00-H59, H92.0, I88.8, I95.1, I95.9, M35.9, M79.5, N30.9, N39.0, N95.1, R03.0, R03.1, R06.2, R06.8, R09.8, R22.4, R25.2, R26.3, R29.6, R29.8, R39.1, R45.2, R49.0, R49.8A, R60.0C, R63.4, R63.5, R77.0, R90.8, R91.9, U99.9, Z00.0, Z00.4, Z03.2, Z03.2A, Z03.8W, Z09.8, Z09.9, Z13.8W, Z13.9, Z22.1, Z22.7, Z59.6, Z59.8, Z59.9, Z63.7, Z72.0, Z73.3, Z73.6, Z73.8, Z73.9, Z82.4, Z85.1, Z85.2A, Z85.3, Z85.4E, Z85.4J, Z85.7, Z85.8C, Z85.8G, Z86.1, Z86.1A, Z88.9, Z92.1, Z97.4 |

**Abbreviations:** COVID-19=Coronavirus disease 2019, ICD-10=International Classification of Diseases 10^th^ revision, PCC=Post COVID-19 condition

**Table S3. Confounders in the adjusted models**

| **Exposure** | **Confounders** |
| --- | --- |
| Education level | Sex, Age category, Born in Sweden |
| Age-standardized income quartile | Sex, Age category, Born in Sweden, Education level, Number of sick days in 2019, Asthma, Cancer, Cardiac/cerebrovascular disease, Chronic kidney failure, Chronic liver disease, Chronic lung disease, Diabetes, Hypertension, Immunocompromised state, Mental health disorder, Neurological disorder, Obesity |
| Number of sick days in 2019 | Sex, Age category, Born in Sweden, Education level, Asthma, Cancer, Cardiac/cerebrovascular disease, Chronic kidney failure, Chronic liver disease, Chronic lung disease, Diabetes, Hypertension, Immunocompromised state, Mental health disorder, Neurological disorder, Obesity |
| Primary care visits in 2019 | Sex, Age category, Born in Sweden, Education level, Age-standardized income quartile, Number of sick days in 2019, Asthma, Cancer, Cardiac/cerebrovascular disease, Chronic kidney failure, Chronic liver disease, Chronic lung disease, Diabetes, Hypertension, Immunocompromised state, Mental health disorder, Neurological disorder, Obesity |
| Outpatient specialist care in 2019 | Sex, Age category, Born in Sweden, Education level, Age-standardized income quartile, Number of sick days in 2019, Asthma, Cancer, Cardiac/cerebrovascular disease, Chronic kidney failure, Chronic liver disease, Chronic lung disease, Diabetes, Hypertension, Immunocompromised state, Mental health disorder, Neurological disorder, Obesity |
| Any inpatient visit in 2019 | Sex, Age category, Born in Sweden, Education level, Age-standardized income quartile, Number of sick days in 2019, Asthma, Cancer, Cardiac/cerebrovascular disease, Chronic kidney failure, Chronic liver disease, Chronic lung disease, Diabetes, Hypertension, Immunocompromised state, Mental health disorder, Neurological disorder, Obesity |
| Asthma | Sex, Age category, Born in Sweden, Education level, Obesity |
| Cancer | Sex, Age category, Born in Sweden, Education level, Obesity |
| Cardiac/cerebrovascular disease | Sex, Age category, Born in Sweden, Education level, Hypertension, Obesity, Diabetes |
| Chronic kidney failure | Sex, Age category, Born in Sweden, Education level, Diabetes, Hypertension, Obesity, Cardiac/cerebrovascular disease |
| Chronic liver disease | Sex, Age category, Born in Sweden, Education level, Diabetes, Hypertension, Obesity, Cardiac/cerebrovascular disease |
| Chronic lung disease | Sex, Age category, Born in Sweden, Education level, Obesity |
| Diabetes | Sex, Age category, Born in Sweden, Education level, Obesity |
| Hypertension | Sex, Age category, Born in Sweden, Education level, Mental health disorder, Obesity |
| Immunocompromised state | Sex, Age category, Born in Sweden, Education level, Cancer, Chronic kidney failure, Chronic liver disease, Diabetes |
| Mental health disorder | Sex, Age category, Born in Sweden, Education level |
| Neurological disorder | Sex, Age category, Born in Sweden, Education level, Cardiac/cerebrovascular disease, Hypertension, Mental health disorder |
| Obesity | Sex, Age category, Born in Sweden, Education level |
| Number of symptoms that could indicate PCC in 2019 | Sex, Age category, Born in Sweden, Education level, Age-standardized income quartile, Number of sick days in 2019, Asthma, Cancer, Cardiac/cerebrovascular disease, Chronic kidney failure, Chronic liver disease, Chronic lung disease, Diabetes, Hypertension, Immunocompromised state, Mental health disorder, Neurological disorder, Obesity |
| COVID-19 vaccination status | Sex, Age category, Born in Sweden, Education level, Age-standardized income quartile, Number of sick days in 2019, Asthma, Cancer, Cardiac/cerebrovascular disease, Chronic kidney failure, Chronic liver disease, Chronic lung disease, Diabetes, Hypertension, Immunocompromised state, Mental health disorder, Neurological disorder, Obesity, SARS-CoV-2 variant, Days since first SARS-CoV-2 infection in Sweden (January 31, 2020), modelled with restricted cubic splines with four knots |
| SARS-CoV-2 variant | Sex, Age category, Born in Sweden, Education level, Age-standardized income quartile, Number of sick days in 2019, Asthma, Cancer, Cardiac/cerebrovascular disease, Chronic kidney failure, Chronic liver disease, Chronic lung disease, Diabetes, Hypertension, Immunocompromised state, Mental health disorder, Neurological disorder, Obesity |

**Abbreviation:** COVID-19=Coronavirus disease 2019, ICU=Intensive care unit, SARS-CoV-2=Severe acute respiratory syndrome coronavirus

**Figure S1. PCC clinic visits over time among non-hospitalized and hospitalized participants**

**Abbreviations:** PCC=Post COVID-19 condition

**Table S4. Adjusted risk ratios vs adjusted subdistribution hazard ratios for PCC clinic attendance among hospitalized participants**

|  | **aRR (95% CI)** | **aSHR (95% CI)** |
| --- | --- | --- |
| Education level ^b^ |  |  |
| Primary | *Reference* | *Reference* |
| Secondary | 0.93 (0.73-1.19) | 0.92 (0.72-1.19) |
| Tertiary | 0.83 (0.64-1.08) | 0.81 (0.63-1.07) |
| Age-standardized income quartile ^c^ |  |  |
| First | *Reference* | *Reference* |
| Second | 1.28 (1.01-1.62) | 1.27 (0.99-1.62) |
| Third | 1.22 (0.92-1.61) | 1.20 (0.90-1.59) |
| Fourth | 0.98 (0.71-1.36) | 0.96 (0.69-1.33) |
| Number of sick days in 2019 ^c^ |  |  |
| 0 | *Reference* | *Reference* |
| 1-30 | 1.56 (1.06-2.29) | 1.57 (1.06-2.33) |
| >31 | 1.94 (1.47-2.56) | 1.97 (1.49-2.62) |
| Primary care visits in 2019 |  |  |
| 0 | *Reference* | *Reference* |
| 1-2 | 1.17 (0.89-1.52) | 1.16 (0.88-1.52) |
| 3-4 | 1.20 (0.87-1.65) | 1.18 (0.86-1.63) |
| >5 | 1.31 (0.95-1.81) | 1.39 (0.93-1.80) |
| Outpatient specialist care in 2019 |  |  |
| 0 | *Reference* | *Reference* |
| 1-2 | 0.95 (0.74-1.22) | 0.94 (0.73-1.22) |
| 3-4 | 0.86 (0.61-1.20) | 0.85 (0.60-1.20) |
| >5 | 1.30 (0.98-1.72) | 1.30 (0.97-1.72) |
| Any inpatient visit in 2019 | 0.83 (0.62-1.11) | 0.83 (0.62-1.12) |
| Comorbidities |  |  |
| Asthma | 1.27 (0.98-1.65) | 1.27 (0.97-1.66) |
| Cancer | 0.89 (0.56-1.39) | 0.91 (0.58-1.44) |
| Cardiac or cerebrovascular disease | 0.71 (0.53-0.97) | 0.73 (0.54-1.00) |
| Chronic kidney failure | 0.78 (0.48-1.27) | 0.80 (0.49-1.31) |
| Chronic liver disease | 0.48 (0.22-1.07) | 0.50 (0.22-1.11) |
| Chronic lung disease | 0.53 (0.31-0.90) | 0.55 (0.32-0.93) |
| Diabetes | 0.99 (0.77-1.27) | 0.99 (0.77-1.28) |
| Hypertension | 1.23 (0.98-1.55) | 1.25 (0.99-1.58) |
| Immunocompromised state | 0.91 (0.65-1.26) | 0.94 (0.67-1.31) |
| Mental health disorder | 1.02 (0.82-1.25) | 1.03 (0.83-1.28) |
| Neurological disorder | 0.19 (0.06-0.61) | 0.20 (0.06-0.64) |
| Obesity | 1.51 (1.19-1.93) | 1.52 (1.19-1.95) |
| Number of symptoms in WHO PCC definition in 2019 |  |  |
| 0 | *Reference* | *Reference* |
| 1 | 1.27 (1.00-1.61) | 1.26 (0.99-1.61) |
| 2 | 1.16 (0.85-1.59) | 1.15 (0.83-1.58) |
| 3 | 1.47 (1.02-2.13) | 1.47 (1.01-2.14) |
| >4 | 0.98 (0.58-1.65) | 0.96 (0.57-1.64) |
| COVID-19 vaccination status |  |  |
| Unvaccinated | *Reference* | *Reference* |
| 1 dose | 0.23 (0.03-1.64) | 0.22 (0.03-1.62) |
| 2 doses | 0.37 (0.10-1.43) | 0.37 (0.10-1.42) |
| 3 doses | 1.01 (0.21-4.89) | 1.00 (0.21-4.84) |
| 4 doses | *Not applicable* | *Not applicable* |
| >5 doses | *Not applicable* | *Not applicable* |
| SARS-CoV-2 variant period |  |  |
| Wild type | *Reference* | *Reference* |
| Alpha | 1.00 (0.81-1.23) | 1.04 (0.84-1.29) |
| Delta | 0.42 (0.24-0.71) | 0.46 (0.27-0.78) |
| Omicron | 0.08 (0.04-0.17) | 0.11 (0.05-0.22) |

**Note:** None of the hospitalized participants who had received 4 or >5 doses of COVID-19 vaccine attended a PCC clinic. None of the non-hospitalized participants who had received >5 doses of COVID-19 vaccine attended a PCC clinic.

**Abbreviations:** aRR=Adjusted risk ratio, aSHR=Adjusted subdistribution hazard ratio, CI=Confidence interval, PCC=Post COVID-19 condition

**Figure S2. Forest plot of baseline characteristics and unadjusted risk ratios of PCC clinic attendance**

**
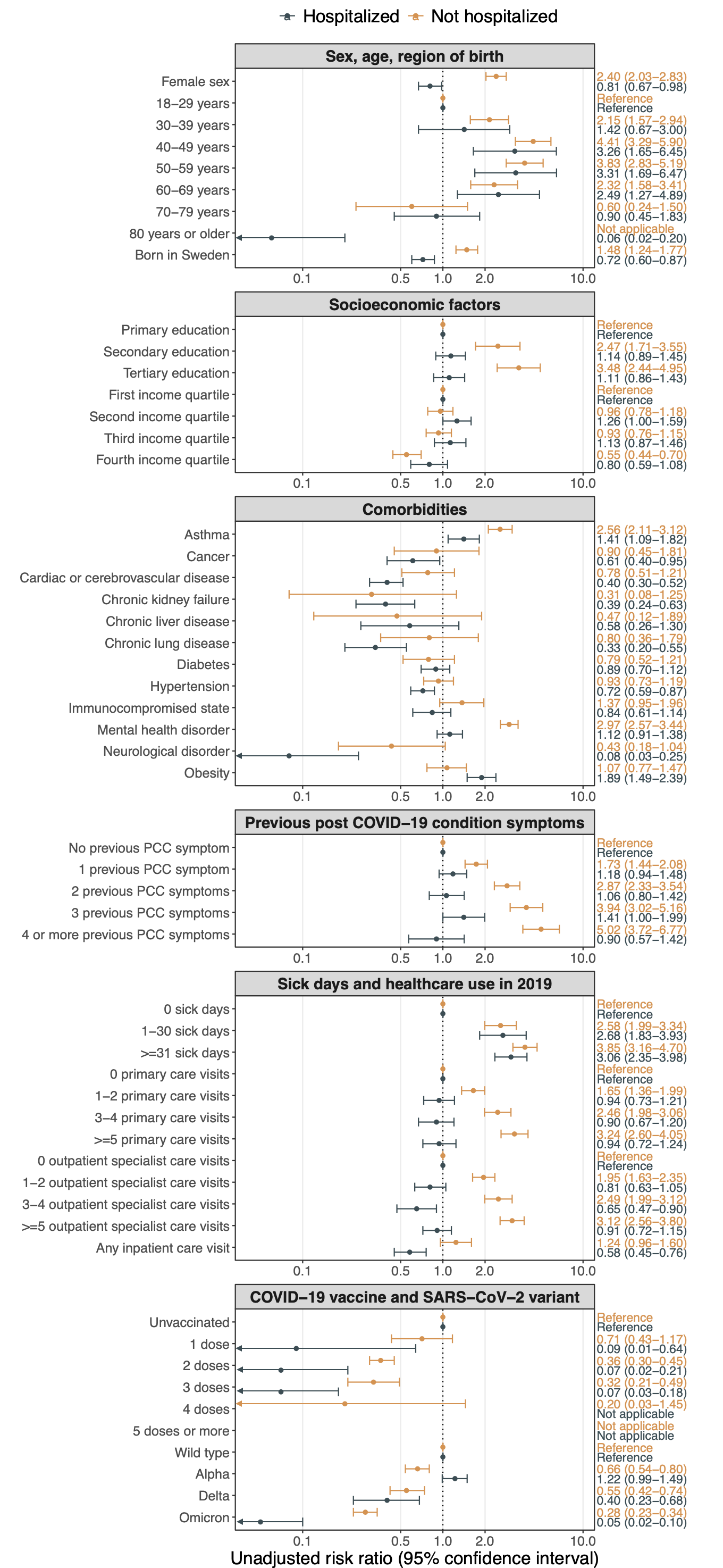
**

**Note:** None of the hospitalized participants who had received ≥4 doses of COVID-19 vaccine attended a PCC clinic. None of the non-hospitalized participants aged 80 years or older or who had received ≥5 doses of COVID-19 vaccine attended a PCC clinic

**Abbreviations:** COVID-19=Coronavirus disease 2019, PCC=Post COVID-19 condition, SARS-CoV-2=Severe acute respiratory syndrome coronavirus 2

**Figure S3. Forest plot of baseline characteristics and adjusted risk ratios of PCC clinic attendance in the main analysis vs the sensitivity analysis among non-hospitalized participants**

**
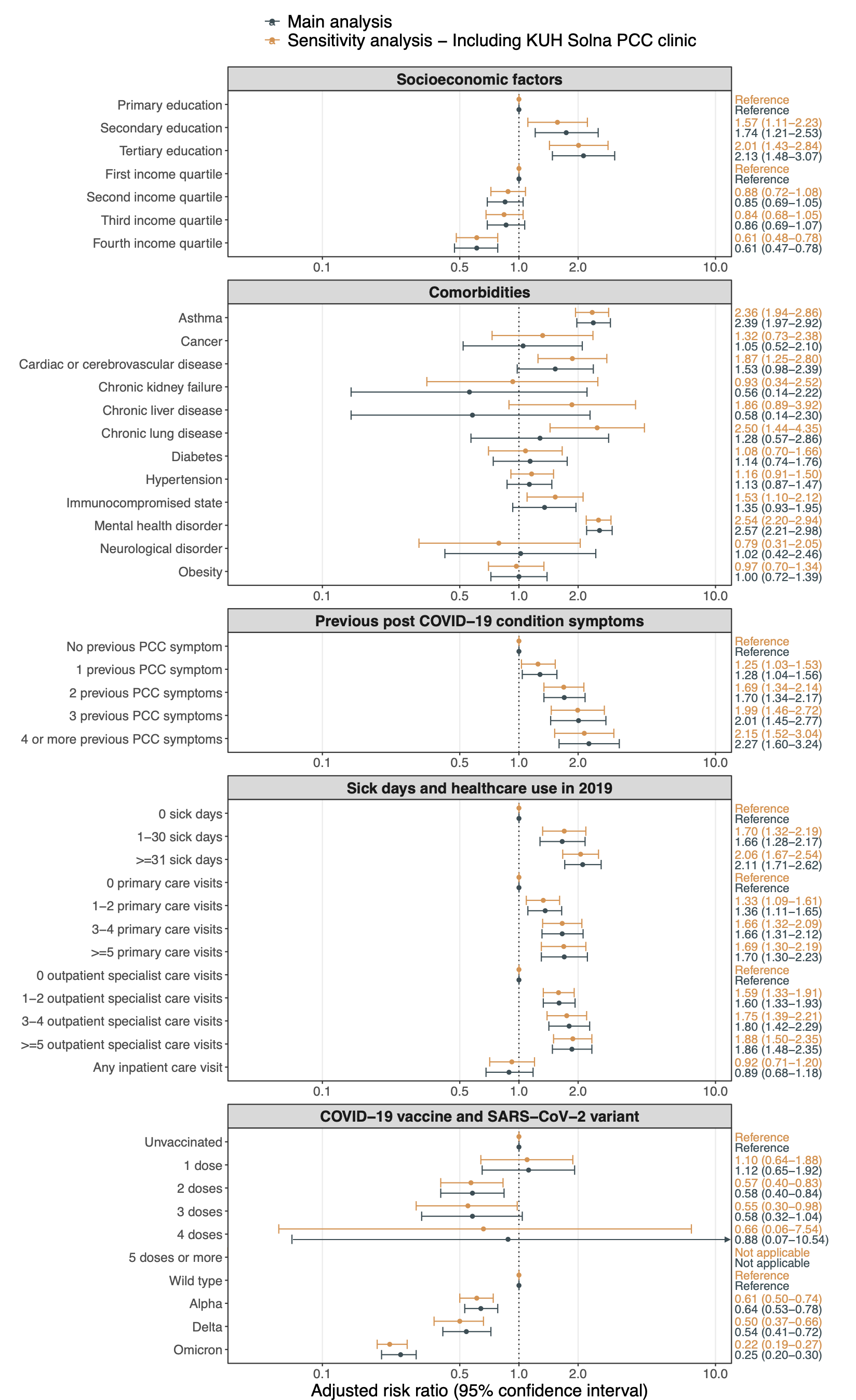
**

**Note:** None of the non-hospitalized who had received ≥5 doses of COVID-19 vaccine attended a PCC clinic

**Abbreviations:** COVID-19=Coronavirus disease 2019, PCC=Post COVID-19 condition, SARS-CoV-2=Severe acute respiratory syndrome coronavirus 2

**Figure S4. Forest plot of baseline characteristics and adjusted risk ratios of PCC clinic attendance in the main analysis vs the sensitivity analysis among hospitalized participants**

**
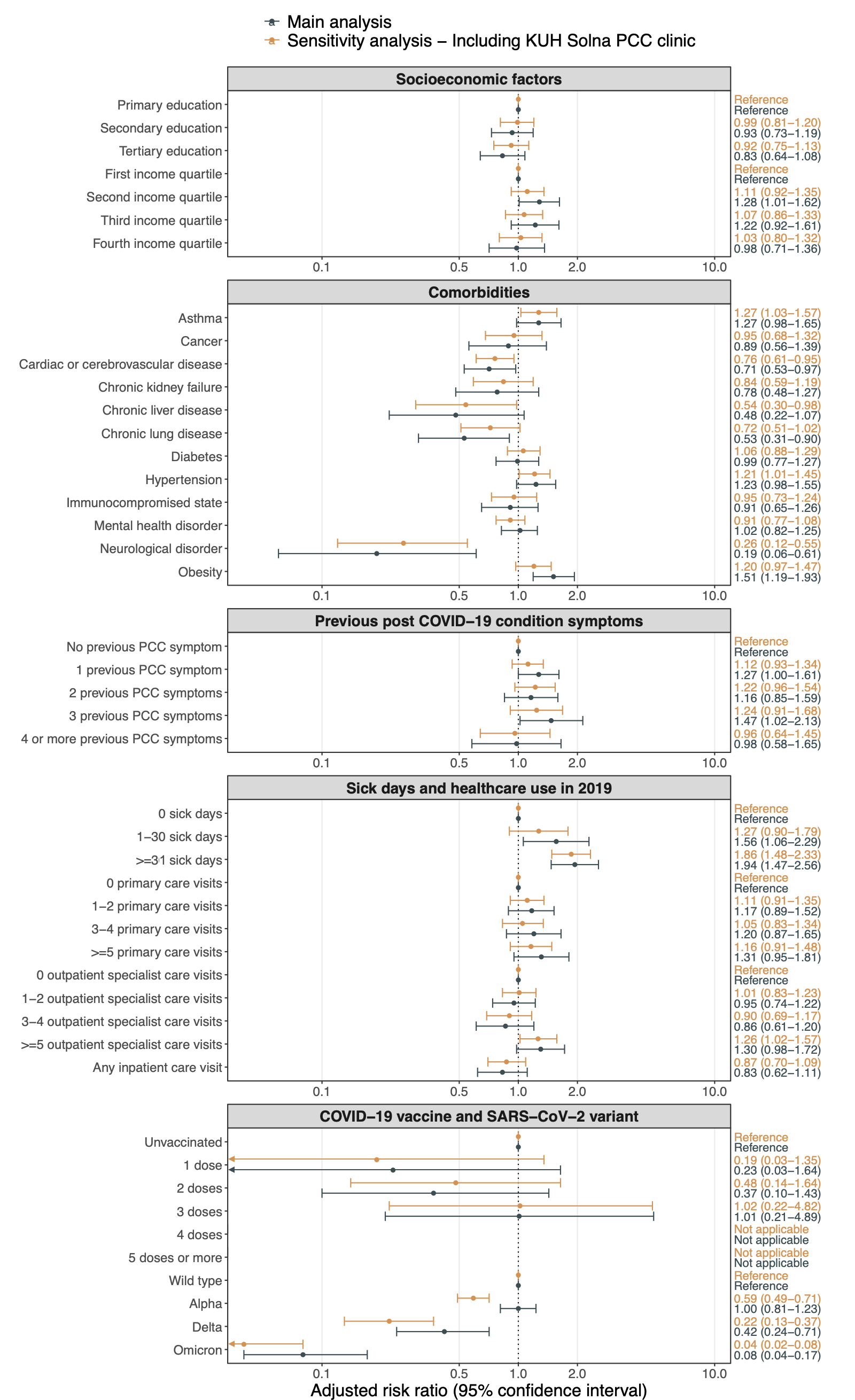
**

**Note:** None of the hospitalized participants who had received ≥4 doses of COVID-19 vaccine attended a PCC clinic.

**Abbreviations:** COVID-19=Coronavirus disease 2019, PCC=Post COVID-19 condition, SARS-CoV-2=Severe acute respiratory syndrome coronavirus 2019

**Figure S5. Forest plot of baseline characteristics and unadjusted risk ratios of PCC clinic attendance among participants with new-onset symptoms that could indicate PCC in primary care**

**
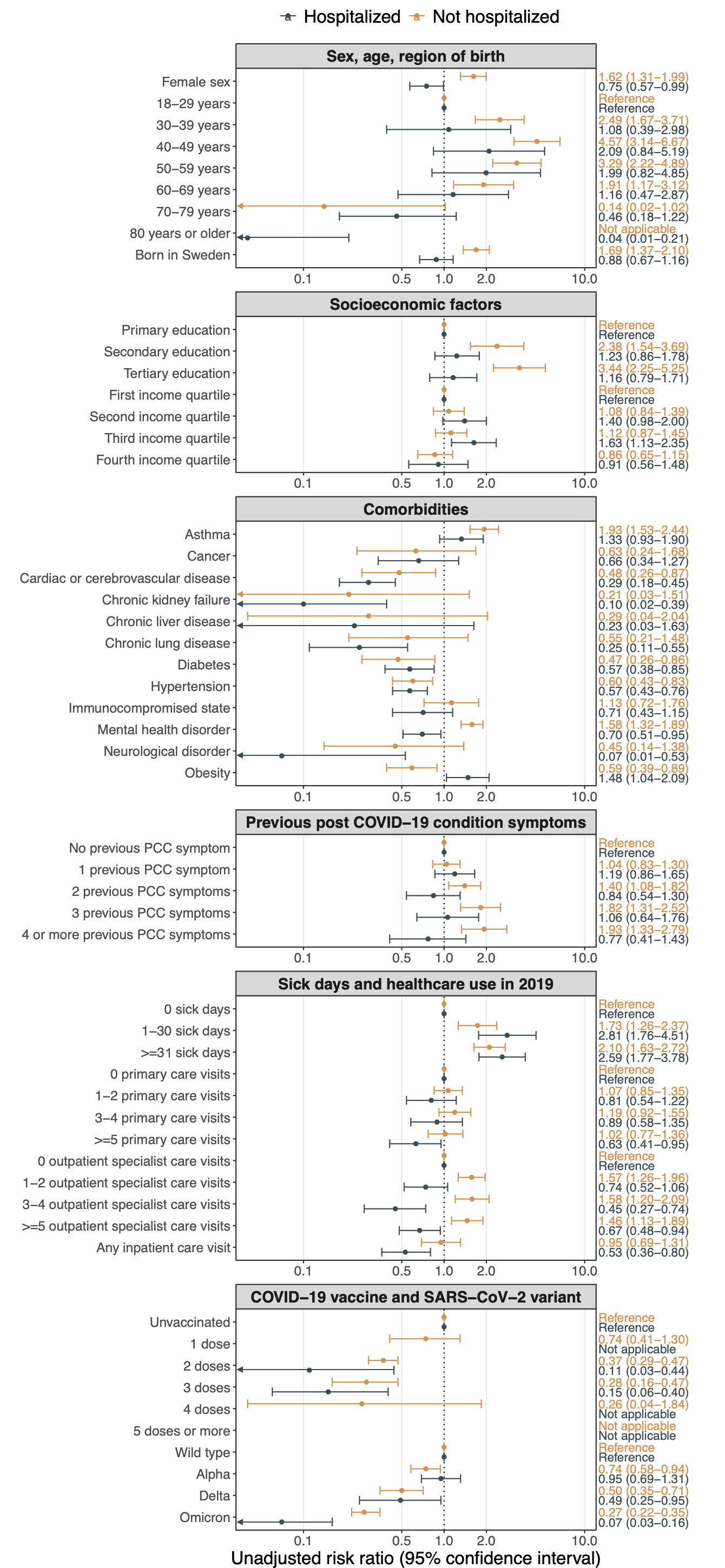
**

**Note:** None of the hospitalized participants who had received ≥4 doses of COVID-19 vaccine attended a PCC clinic. None of the non-hospitalized participants aged 80 years or older or who had received ≥5 doses of COVID-19 vaccine attended a PCC clinic.

**Abbreviations:** COVID-19=Coronavirus disease 2019, PCC=Post COVID-19 condition, SARS-CoV-2=Severe acute respiratory syndrome coronavirus 2

**Figure S6. Forest plot of baseline characteristics and adjusted risk ratios of PCC clinic attendance among participants with new-onset symptoms that could indicate PCC in primary care**


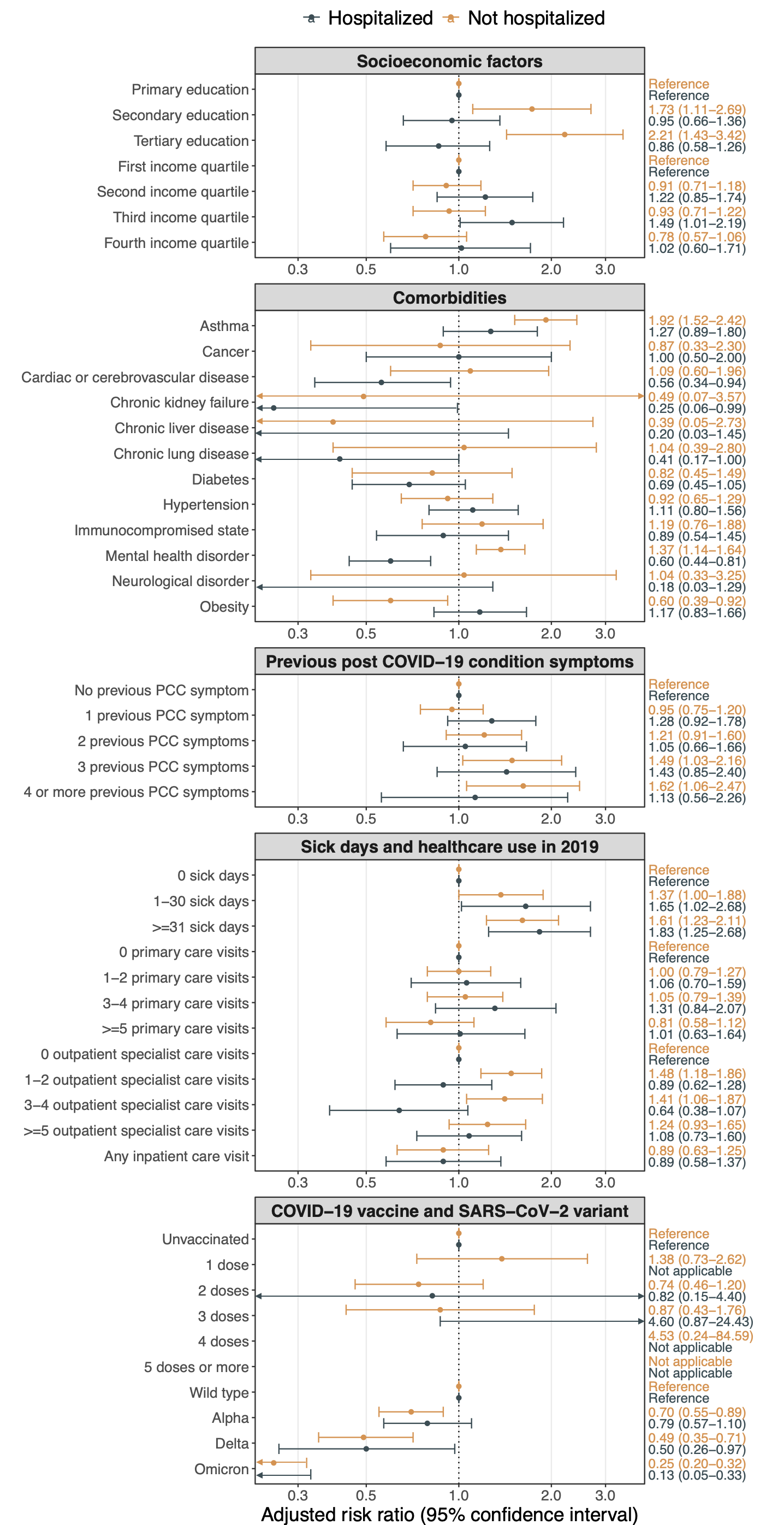


**Notes:** Cofounders for each model are described in Table S3. None of the hospitalized participants who had received ≥4 doses of COVID-19 vaccine attended a PCC clinic. None of the non-hospitalized participants who had received >5 doses of COVID-19 vaccine attended a PCC clinic.

**Abbreviations:** COVID-19=Coronavirus disease 2019, PCC=Post COVID-19 condition, SARS-CoV-2=Severe acute respiratory syndrome coronavirus 2

**Table S5. Comparison of participants with a PCC diagnosis registered in primary care and participants attending a PCC clinic**

|  | **Not hospitalized (n=3657)** | | | **Hospitalized  (n=1467)** | | |
| --- | --- | --- | --- | --- | --- | --- |
| **Variable** | **Primary care (n=2920)** | **PCC clinic (n=737)** | ***P*** | **Primary care (n=1034)** | **PCC clinic (n=433)** | ***P*** |
| Female sex | 2031 (69.6) | 548 (74.4) | .01 | 470 (45.5) | 173 (40.0) | .06 |
| Age, years | 48.0 [39.0, 56.0] | 46.0 [39.0, 52.0] | <.001 | 60.0 [52.0, 70.0] | 56.0 [48.0, 64.0] | <.001 |
| Age category, years |  |  | <.001 |  |  | <.001 |
| 18-29 | 194 (6.6) | 53 (7.2) |  | 10 (1.0) | 9 (2.1) |  |
| 30-39 | 564 (19.3) | 142 (19.3) |  | 55 (5.3) | 28 (6.5) |  |
| 40-49 | 833 (28.5) | 293 (39.8) |  | 141 (13.6) | 88 (20.3) |  |
| 50-59 | 853 (29.2) | 193 (26.2) |  | 298 (28.8) | 139 (32.1) |  |
| 60-69 | 361 (12.4) | 51 (6.9) |  | 261 (25.2) | 115 (26.6) |  |
| 70-79 | 94 (3.2) | 5 (0.7) |  | 189 (18.3) | 50 (11.5) |  |
| >80 | 21 (0.7) | 0 |  | 80 (7.7) | 4 (0.9) |  |
| Born in Sweden ^a^ | 2005 (68.7) | 588 (79.8) | <.001 | 574 (55.5) | 224 (51.7) | .20 |
| Education level ^b^ |  |  | <.001 |  |  | .15 |
| Primary | 242 (8.3) | 33 (4.5) |  | 182 (17.9) | 94 (22.2) |  |
| Secondary | 1070 (36.8) | 235 (32.0) |  | 446 (43.9) | 181 (42.8) |  |
| Tertiary | 1593 (54.8) | 467 (63.5) |  | 387 (38.1) | 148 (35.0) |  |
| Age-standardized income quartile |  |  | .02 |  |  | .27 |
| First | 552 (18.9) | 150 (20.4) |  | 286 (27.7) | 133 (30.7) |  |
| Second | 1025 (35.1) | 223 (30.3) |  | 313 (30.3) | 139 (32.1) |  |
| Third | 763 (26.1) | 228 (30.9) |  | 252 (24.4) | 100 (23.1) |  |
| Fourth | 580 (19.9) | 136 (18.5) |  | 183 (17.7) | 61 (14.1) |  |
| Number of sick days in 2019 |  |  | .004 |  |  | .04 |
| 0 | 2322 (79.5) | 556 (75.4) |  | 862 (83.4) | 343 (79.2) |  |
| 1-30 | 267 (9.1) | 64 (8.7) |  | 69 (6.7) | 27 (6.2) |  |
| >31 | 331 (11.3) | 117 (15.9) |  | 103 (10.0) | 63 (14.5) |  |
| Primary care visits in 2019 |  |  | .10 |  |  | .75 |
| 0 | 637 (21.8) | 182 (24.7) |  | 202 (19.5) | 95 (21.9) |  |
| 1-2 | 1041 (35.7) | 272 (36.9) |  | 363 (35.1) | 144 (33.3) |  |
| 3-4 | 596 (20.4) | 147 (19.9) |  | 200 (19.3) | 82 (18.9) |  |
| >5 | 646 (22.1) | 136 (18.5) |  | 269 (26.0) | 112 (25.9) |  |
| Outpatient specialist care in 2019 |  |  | .01 |  |  | .01 |
| 0 | 1105 (37.8) | 232 (31.5) |  | 306 (29.6) | 148 (34.2) |  |
| 1-2 | 816 (27.9) | 224 (30.4) |  | 293 (28.3) | 99 (22.9) |  |
| 3-4 | 425 (14.6) | 112 (15.2) |  | 153 (14.8) | 48 (11.1) |  |
| >5 | 574 (19.7) | 169 (22.9) |  | 282 (27.3) | 138 (31.9) |  |
| Any inpatient visit in 2019 | 219 (7.5) | 64 (8.7) | .32 | 165 (16.0) | 65 (15.0) | .71 |
| Comorbidities |  |  |  |  |  |  |
| Asthma | 383 (13.1) | 119 (16.1) | .04 | 191 (18.5) | 66 (15.2) | .16 |
| Cancer | 31 (1.1) | 8 (1.1) | >.99 | 48 (4.6) | 21 (4.8) | .97 |
| Cardiac or cerebrovascular disease | 129 (4.4) | 21 (2.8) | .07 | 166 (16.1) | 61 (14.1) | .38 |
| Chronic kidney failure | 20 (0.7) | 2 (0.3) | .30 | 56 (5.4) | 18 (4.2) | .38 |
| Chronic liver disease | 17 (0.6) | 2 (0.3) | .45 | 19 (1.8) | 6 (1.4) | .70 |
| Chronic lung disease | 50 (1.7) | 6 (0.8) | .11 | 72 (7.0) | 16 (3.7) | .02 |
| Diabetes | 140 (4.8) | 22 (3.0) | .04 | 217 (21.0) | 86 (19.9) | .68 |
| Hypertension | 410 (14.0) | 69 (9.4) | <.001 | 416 (40.2) | 167 (38.6) | .59 |
| Immunocompromised state | 115 (3.9) | 31 (4.2) | .82 | 98 (9.5) | 43 (9.9) | .86 |
| Mental health disorder | 1326 (45.4) | 376 (51.0) | .007 | 344 (33.3) | 125 (28.9) | .11 |
| Neurological disorder | 12 (0.4) | 5 (0.7) | .52 | 30 (2.9) | 3 (0.7) | .02 |
| Obesity | 240 (8.2) | 39 (5.3) | .009 | 143 (13.8) | 82 (18.9) | .02 |
| Number of symptoms in WHO PCC definition in 2019 |  |  | .75 |  |  | .22 |
| 0 | 1252 (42.9) | 321 (43.6) |  | 450 (43.5) | 197 (45.5) |  |
| 1 | 773 (26.5) | 181 (24.6) |  | 259 (25.0) | 120 (27.7) |  |
| 2 | 469 (16.1) | 122 (16.6) |  | 166 (16.1) | 58 (13.4) |  |
| 3 | 260 (8.9) | 64 (8.7) |  | 85 (8.2) | 38 (8.8) |  |
| >4 | 166 (5.7) | 49 (6.6) |  | 74 (7.2) | 20 (4.6) |  |
| COVID-19 vaccination status |  |  | .008 |  |  | .002 |
| Unvaccinated | 2488 (85.2) | 589 (79.9) |  | 962 (93.0) | 425 (98.2) |  |
| 1 dose | 40 (1.4) | 16 (2.2) |  | 11 (1.1) | 1 (0.2) |  |
| 2 doses | 311 (10.7) | 110 (14.9) |  | 34 (3.3) | 3 (0.7) |  |
| 3 doses | 78 (2.7) | 21 (2.8) |  | 18 (1.7) | 4 (0.9) |  |
| 4 doses | 3 (0.1) | 1 (0.1) |  | 9 (0.9) | 0 |  |
| >5 doses | 2488 (85.2) | 589 (79.9) |  | 0 | 0 |  |
| SARS-CoV-2 variant period |  |  | .001 |  |  | <.001 |
| Wild type | 1735 (59.4) | 422 (57.3) |  | 572 (55.3) | 279 (64.4) |  |
| Alpha | 642 (22.0) | 133 (18.0) |  | 316 (30.6) | 132 (30.5) |  |
| Delta | 170 (5.8) | 53 (7.2) |  | 84 (8.1) | 14 (3.2) |  |
| Omicron | 373 (12.8) | 129 (17.5) |  | 62 (6.0) | 8 (1.8) |  |
| ICU admitted with COVID-19 | NA | NA | NA | 266 (25.7) | 250 (57.7) | <.001 |
| Reason for end of follow-up |  |  | .07 |  |  | .003 |
| Administrative | 2831 (97.0) | 726 (98.5) |  | 974 (94.2) | 425 (98.2) |  |
| Death | 9 (0.3) | 1 (0.1) |  | 44 (4.3) | 4 (0.9) |  |
| Moving out of Stockholm County | 80 (2.7) | 10 (1.4) |  | 16 (1.5) | 4 (0.9) |  |

a. Data were missing for 1 participant who was excluded from these analyses

b. Data were missing for 46 participants who were excluded from these analyses

**Note:** Data are presented as numbers and percentages or medians and interquartile intervals. Mann-Whitney U tests were used for continuous variables and Chi-squared tests were used for categorical variables.

**Abbreviations:** COVID-19=Coronavirus disease 2019, ICU=Intensive care unit, NA=Not applicable, PCC=Post COVID-19 condition, SARS-CoV-2=Severe acute respiratory syndrome coronavirus 2
