# Supplementary material for: Determinants of post COVID-19 clinic attendance among SARS-CoV-2-infected individuals in Stockholm, Sweden: a population-based cohort study
Source: BMJ Open. 2025 Jun 17;15(6):e098344. doi: 10.1136/bmjopen-2024-098344 (PMC12182103; doi:10.1136/bmjopen-2024-098344)
Supplement: Supplementary file 2 [file bmjopen-2024-098344.reviewer_comments.pdf]

PEER REVIEW HISTORY

BMJ Open publishes all reviews undertaken for accepted manuscripts. Reviewers are asked to complete a checklist review form and are provided with free text boxes to elaborate on their assessment. These free text comments are reproduced below.

ARTICLE DETAILS

Title (Provisional)

Determinants of post COVID-19 clinic attendance among SARS-CoV-2 infected individuals in Stockholm, Sweden: A population-based cohort study

Authors

Hedberg, Pontus; af Geijerstam, Peder; Karlsson Valik, John; Almgren-Lidman, Christer; Ternhag, Anders; Naucler, Pontus

---

VERSION 1 - REVIEW

**Reviewer**	**1**
**Name**	**Mumoli, Nicola**
**Affiliation**	**Department of Internal Medicine, Magenta Hospital, Italy**
**Date**	**09-Apr-2025**
**COI**	**None**

This is a well-executed population-based cohort study using linked registry data from Stockholm County, Sweden, to investigate determinants of post-COVID-19 condition (PCC) clinic attendance.

The authors, however, should carefully address all the points raised above and provide a point-by-point response in their revision:

1) The authors rely on symptom codes and clinic attendance to define PCC. Given the known limitations of the WHO clinical case definition (noted by the authors), further discussion on possible misclassification (e.g., symptoms unrelated to PCC) would be valuable; you add a short discussion on the implications of false positives and specificity issues in PCC coding.

2) The study identifies "new-onset" symptoms in primary care but acknowledges difficulty distinguishing PCC from other conditions; please clarify how symptoms were attributed to PCC and if any clinical adjudication or criteria beyond ICD codes were applied.

3) While the paper discusses education and health literacy, more elaboration is needed on how systemic barriers (e.g., referral patterns, socioeconomic constraints) may have influenced access to PCC clinics; please expand discussion of potential structural determinants of care inequity.

4) More detail is needed on how vaccination status was integrated, especially for infections occurring in 2021-2022. Were any time-to-event analyses performed accounting for timing of vaccination? you briefly clarify the temporal relationship between vaccination and infection in the methods.

5) Minor copyediting to tighten wording in the discussion would improve clarity.

6) Tables and Figures: Clear and informative. Figure 2 (forest plot) may benefit from bolder group headings (e.g., hospitalized vs non-hospitalized). Supplementary Materials: Ensure that references to Tables S1-S5 and Figures S1-S6 match consistently throughout the manuscript.

**Reviewer**	**2**
**Name**	**Zeng , Guangting**
**Affiliation**	**Xiangtan University**
**Date**	**29-Apr-2025**
**COI**	**None**

This large population-based cohort study examined differences in symptoms and factors influencing presentation to the PCC clinic between patients who were hospitalized and those who were not during acute SARS-CoV-2 infection.This study could help healthcare decision makers understand the demand and heterogeneity of PCC care among patients with long COVID.Here, I have a few suggestions to add.

First,the study did not collect information about treatments (medications, exercise rehabilitation, etc.) that patients received for COVID-19. It focused only on PCC clinic visits and did not involve any interventions or assessments of treatment effects. Also, this study did not assess the duration or severity of PCC symptoms in detail, which is critical to understanding the long-term effects of PCC and developing treatment strategies. These limitations may limit the clinical value of this study.

Second, while the determinants of PCC clinic visits and heterogeneity in clinical presentation are discussed, more specific recommendations are lacking on how to improve diagnostic and treatment strategies for PCC, and how to reduce the risk of resource mismatch and inappropriate care.

Third, thw number of PCC outpatient visits may be related to the publicity of long COVID-19 knowledge and the availability of medical services, which needs to be considered.

VERSION 1 - AUTHOR RESPONSE

Reviewer 1

This is a well-executed population-based cohort study using linked registry data from Stockholm County, Sweden, to investigate determinants of post-COVID-19 condition (PCC) clinic attendance.

Reply: We would like to thank Reviewer 1 for the thorough and constructive review of our manuscript and for the kind comment with regards to our study.

The authors, however, should carefully address all the points raised above and provide a point-by-point response in their revision:

1) The authors rely on symptom codes and clinic attendance to define PCC. Given the known limitations of the WHO clinical case definition (noted by the authors), further discussion on possible misclassification (e.g., symptoms unrelated to PCC) would be valuable; you add a short discussion on the implications of false positives and specificity issues in PCC coding.

Reply: We agree with Reviewer 1 on the importance of further addressing possible outcome misclassification, given the known limitations of the WHO clinical case definition for PCC. This definition has limited specificity, especially for symptoms prevalent in the general population (e.g., fatigue, dyspnoea). Accordingly, we have added the following in the revised Discussion:

"Furthermore, due to the overlap between PCC symptoms and those of other conditions, the use of ICD-coded symptoms and clinic attendance may have introduced outcome misclassification. Importantly, symptom codes registered in primary care or during PCC-related visits may not necessarily reflect PCC. This may result in both false positive and false negative classifications, particularly in the absence of standardized clinical adjudication. Future studies incorporating objective functional assessments and standardized diagnostic criteria are needed to improve PCC case ascertainment."

2) The study identifies "new-onset" symptoms in primary care but acknowledges difficulty distinguishing PCC from other conditions; please clarify how symptoms were attributed to PCC and if any clinical adjudication or criteria beyond ICD codes were applied.

Reply: We used time-bound criteria (28 through 180 days after the first positive PCR or serology) and excluded individuals with records of the same symptoms in 2019 to minimise misattribution. These analyses were based on ICD-10 codes, without manual adjudication. To clarify this, we have updated the Methods section:

"To understand determinants of PCC clinic attendance after a primary care presentation which could indicate PCC, such a cohort was defined. Individuals with any of 19 out of 25

symptoms included in the World Health Organization (WHO) PCC case definition (Table S1) registered in primary care from 28 through 180 days after the first positive PCR or serology result, that did not have a history of the symptom(s) in 2019, were included [7]. Symptoms were identified by registered ICD-10 codes. The 6 symptoms blurred vision, cognitive dysfunction, memory issues, menstrual problems, allergies, and post-exertional malaise were excluded due to insufficient coverage in the available data, as previously described [5].”

3) While the paper discusses education and health literacy, more elaboration is needed on how systemic barriers (e.g., referral patterns, socioeconomic constraints) may have influenced access to PCC clinics; please expand discussion of potential structural determinants of care inequity.

Reply: We would like to thank Reviewer 1 for addressing this important point. We have elaborated on this in the Discussion as follows:

“Importantly, access to PCC services may be influenced by systemic barriers such as referral practices, language, and socioeconomic constraints. The observed association between higher education and Swedish birth with PCC clinic attendance suggest inequities in access to care. These disparities may reflect differences in health literacy, capacity to navigate the healthcare system, or implicit bias in referral decisions.”

4) More detail is needed on how vaccination status was integrated, especially for infections occurring in 2021-2022. Were any time-to-event analyses performed accounting for timing of vaccination? you briefly clarify the temporal relationship between vaccination and infection in the methods.

Reply: We thank the reviewer for this valuable comment. While we captured vaccination status before infection, we did not perform time-to-event analyses. We have updated the Methods section to clarify this as follows:

“COVID-19 vaccination status was categorized as unvaccinated, or as having received 1, 2, 3, 4 or 5 or more doses administered at least 14 days before the acute SARS-CoV-2 infection. We did not adjust for the exact timing between vaccination and infection (see Table S1 for more details).”

5) Minor copyediting to tighten wording in the discussion would improve clarity.

Reply: We agree with the reviewer and have revised the Discussion for clarity, removed redundancies, and streamlined language.

6) Tables and Figures: Clear and informative. Figure 2 (forest plot) may benefit from bolder group headings (e.g., hospitalized vs non-hospitalized). Supplementary Materials: Ensure that references to Tables S1-S5 and Figures S1-S6 match consistently throughout the manuscript.

Reply: We would like to thank Reviewer 1 for address these points. We have bolded group headings in Figure 3 (forest plot) for readability and verified consistency of references to Tables S1–S6 and Figures S1–S6. This includes having corrected *"Low levels of missing data were observed for region of birth, education level, age-standardized income quartile, and number of sick days in 2019 (Table **S2**)."* to *"Low levels of missing data were observed for region of birth, education level, age-standardized income quartile, and number of sick days in 2019 (Table **S1**)."*

Reviewer 2

This large population-based cohort study examined differences in symptoms and factors influencing presentation to the PCC clinic between patients who were hospitalized and those who were not during acute SARS-CoV-2 infection. This study could help healthcare decision makers understand the demand and heterogeneity of PCC care among patients with long COVID. Here, I have a few suggestions to add.

Reply: We would like to thank Reviewer 2 for the thorough and constructive review of our manuscript. We agree with the Reviewer that these findings are of clinical importance in order to facilitate better PCC-related care and to highlight potential sociodemographic disparities in PCC-related service utilization.

First, the study did not collect information about treatments (medications, exercise rehabilitation, etc.) that patients received for COVID-19. It focused only on PCC clinic visits and did not involve any interventions or assessments of treatment effects. Also, this study did not assess the duration or severity of PCC symptoms in detail, which is critical to understanding the long-term effects of PCC and developing treatment strategies. These limitations may limit the clinical value of this study.

Reply: We would like to thank Reviewer 2 for addressing these important points. We completely agree on this limitation, it was not the scope of the study. However, we have updated the Discussion section accordingly:

"Importantly, this study did not include data on treatments received (e.g., rehabilitation, medication), nor symptom severity or duration. These factors are critical to understanding recovery trajectories and treatment effects. Inclusion of such data should be prioritized in future longitudinal studies."

Second, while the determinants of PCC clinic visits and heterogeneity in clinical presentation are discussed, more specific recommendations are lacking on how to improve diagnostic and treatment strategies for PCC, and how to reduce the risk of resource mismatch and inappropriate care.

Reply: Thank you for highlighting this important point. We agree that formulating evidence-based recommendations is a key next step to improve PCC care. While our study is observational and cannot directly inform diagnostic or therapeutic strategies, our findings highlight the variability in PCC presentation and access to care, suggesting a need for stratified, patient-centered care models. Future research should prioritize the development and validation of specific diagnostic criteria, referral pathways, and scalable care strategies that can minimize inappropriate utilization while ensuring access for underserved populations. We have expanded the Conclusion to reflect these implications.

"*Future research should explore stratified care models and targeted interventions to improve equity and guide appropriate service provision.*"

Third, the number of PCC outpatient visits may be related to the publicity of long COVID-19 knowledge and the availability of medical services, which needs to be considered.

Reply: We completely agree with the reviewer and have added the following to the Discussion:

"Clinic attendance likely reflects not only clinical need but also awareness and availability. Media attention, public concern, and clinic capacity during early pandemic waves may have influenced PCC identification. Such contextual factors should be considered when interpreting absolute attendance rates."

VERSION 2 - REVIEW

**Reviewer**	**1**
**Name**	**Mumoli, Nicola**
**Affiliation**	**Department of Internal Medicine, Magenta Hospital, Italy**
**Date**	**26-May-2025**
**COI**	

The authors have sufficiently addressed all the comments by the reviewers.
